# Supplementary material for: Diversity of major histocompatibility complex of II B gene and mate choice in a monogamous and long-lived seabird, the Little Auk (Alle alle)
Source: PLoS One. 2024 Jun 12;19(6):e0304275. doi: 10.1371/journal.pone.0304275 (PMC11168636; doi:10.1371/journal.pone.0304275)
Supplement: S1 Table — (DOCX) [file pone.0304275.s001.docx]

**Supplementary materials**

**S1 Table.** Sequences of primers that identify exon 2 MHC in the Little Auk not used for further genotyping as provided longer products, not appropriate for Ion Torrent sequencing.

| Primer name | Sequence 5’ - 3’ |
| --- | --- |
| AukLz2 | TGCAGATBCCCTGGGGGCAGCTG |
| Alal2L | TGCAGGAGGATGCTGTGMAGGRAGGA |
| Gal5L | CAGCAACGGCACHRAGCGBGTGAGG |
| Alal1R | GGGKAGAYYTYCACCTYGGGCT |
| Alal2R | GGCAGGGAGCTCGACTGCABBG |
| AukRz1 | GGRCTGCCCRGGGCTTGGCTTG |
| AukRz2 | CTCCCTCTGCTCCCYRCGCAGGACGT |
| AukRw1 | TAGTTGTTTCGGCAGWSCGTGTCCA |
