# Supplementary material for: Diversity of major histocompatibility complex of II B gene and mate choice in a monogamous and long-lived seabird, the Little Auk (Alle alle)
Source: PLoS One. 2024 Jun 12;19(6):e0304275. doi: 10.1371/journal.pone.0304275 (PMC11168636; doi:10.1371/journal.pone.0304275)
Supplement: S2 Table — (DOCX) [file pone.0304275.s002.docx]

**Supplementary materials**

**S2 Table**. List of barcodes used in bidirectional tagging for Ion Torrent PGM sequencing. The sample-specific barcode primer pairs comprised of the Ion Torrent adapter sequences (A and P1) appended to 10-bp sequence tags:

**A** (-CCATCTCATCCCTGCGTGTCTCCGACTCAG-) + Barcode(A01-A16) + linker (-GAT-) + primer *Forward* (-ATGTCTGCMCGAGCAGGGWA-)

**P1** (-CCTCTCTATGGGCAGTCGGTGATG-) + Barcode (P17-P32) + linker (-AT-) + primer *Revers* (-CCRGGGCTTGGCTTGTGCCTG-);

| Barcode | Sequence |
| --- | --- |
| A01 | CTAAGGTAAC |
| A02 | TAAGGAGAAC |
| A03 | AAGAGGATTC |
| A04 | TACCAAGATC |
| A05 | CAGAAGGAAC |
| A06 | CTGCAAGTTC |
| A07 | TTCGTGATTC |
| A08 | TTCCGATAAC |
| A09 | TGAGCGGAAC |
| A10 | CTGACCGAAC |
| A11 | TCCTCGAATC |
| A12 | TAGGTGGTTC |
| A13 | TCTAACGGAC |
| A14 | TTGGAGTGTC |
| A15 | TCTAGAGGTC |
| A16 | TCTGGATGAC |
| P17 | ACGAATAGAG |
| P18 | CAATTGCCTG |
| P19 | TCCGACTAAG |
| P20 | ATGGATCTGG |
| P21 | TAATTGCGAG |
| P22 | CGTCTCGAAG |
| P23 | TTCGTGGCAG |
| P24 | AATGAGGTTG |
| P25 | TATCTCAGGG |
| P26 | AGGTTGTAAG |
| P27 | CGGATGGTTG |
| P28 | ATTCCGGATG |
| P29 | AGTGGTCGAG |
| P30 | ATAACCTCGG |
| P31 | CAGCTTGGAG |
| P32 | TGTGTAAGAG |
