# Supplementary material for: Diversity of major histocompatibility complex of II B gene and mate choice in a monogamous and long-lived seabird, the Little Auk (Alle alle)
Source: PLoS One. 2024 Jun 12;19(6):e0304275. doi: 10.1371/journal.pone.0304275 (PMC11168636; doi:10.1371/journal.pone.0304275)
Supplement: S3 Table — (DOCX) [file pone.0304275.s003.docx]

**Supplementary materials**

**S3 Table**. Full list of alleles with their depth and frequency in the sampled individuals (n = 140).

| **Allele Name (GeneBank)** | **Allele Name (in the manuscript figures)** | **Depth** | **N ind with the allele** |
| --- | --- | --- | --- |
| MHCII-0000001-C | 1 | 44181 | 47 |
| MHCII-0000005-C | 2 | 14119 | 20 |
| MHCII-0000006-C | 3 | 11394 | 19 |
| MHCII-0000003-C | 4 | 17887 | 13 |
| MHCII-0000010-C | 5 | 7821 | 11 |
| MHCII-0000013-C | 6 | 4104 | 8 |
| MHCII-0000015-C | 7 | 7030 | 7 |
| MHCII-0000009-C | 8 | 5664 | 6 |
| MHCII-0000027-C | 15 | 3292 | 6 |
| MHCII-0000004-C | 9 | 8055 | 4 |
| MHCII-0000008-C | 10 | 6039 | 4 |
| MHCII-0000019-C | 11 | 3374 | 4 |
| MHCII-0000022-C | 12 | 3118 | 4 |
| MHCII-0000024-C | 13 | 2969 | 4 |
| MHCII-0000029-C | 14 | 2643 | 4 |
| MHCII-0000041-C | 16 | 2363 | 4 |
| MHCII-0000011-C | 17 | 4825 | 3 |
| MHCII-0000025-C | 18 | 2911 | 3 |
| MHCII-0000026-C | 19 | 2901 | 3 |
| MHCII-0000034-C | 20 | 2897 | 3 |
| MHCII-0000035-C | 21 | 2049 | 3 |
| MHCII-0000036-C | 22 | 2025 | 3 |
| MHCII-0000042-C | 23 | 1902 | 3 |
| MHCII-0000044-C | 24 | 1789 | 3 |
| MHCII-0000056-C | 37 | 1537 | 3 |
| MHCII-0000051-C | 38 | 1655 | 3 |
| MHCII-0000014-C | 25 | 3826 | 2 |
| MHCII-0000018-C | 26 | 3375 | 2 |
| MHCII-0000020-C | 29 | 3343 | 2 |
| MHCII-0000028-C | 30 | 2699 | 2 |
| MHCII-0000030-C | 31 | 2608 | 2 |
| MHCII-0000031-C | 32 | 2469 | 2 |
| MHCII-0000039-C | 33 | 1941 | 2 |
| MHCII-0000043-C | 34 | 1865 | 2 |
| MHCII-0000047-C | 35 | 1723 | 2 |
| MHCII-0000052-C | 36 | 1399 | 2 |
| MHCII-0000095-C | 39 | 1060 | 2 |
| MHCII-0000070-C | 40 | 995 | 2 |
| MHCII-0000072-C | 41 | 939 | 2 |
| MHCII-0000075-C | 42 | 918 | 2 |
| MHCII-0000058-C | 43 | 819 | 2 |
| MHCII-0000093-C | 44 | 678 | 2 |
| MHCII-0000094-C | 45 | 638 | 2 |
| MHCII-0000097-C | 46 | 620 | 2 |
| MHCII-0000053-C | 57 | 1857 | 2 |
| MHCII-0000069-C | 27 | 997 | 1 |
| MHCII-0000033-C | 28 | 2365 | 1 |
| MHCII-0000112-C | 47 | 285 | 1 |
| MHCII-0000016-C | 48 | 3537 | 1 |
| MHCII-0000021-C | 49 | 3327 | 1 |
| MHCII-0000032-C | 50 | 2408 | 1 |
| MHCII-0000037-C | 51 | 2013 | 1 |
| MHCII-0000038-C | 52 | 1976 | 1 |
| MHCII-0000045-C | 53 | 1781 | 1 |
| MHCII-0000048-C | 54 | 1717 | 1 |
| MHCII-0000049-C | 55 | 1632 | 1 |
| MHCII-0000050-C | 56 | 1604 | 1 |
| MHCII-0000054-C | 58 | 1342 | 1 |
| MHCII-0000055-C | 59 | 1314 | 1 |
| MHCII-0000057-C | 60 | 1267 | 1 |
| MHCII-0000060-C | 61 | 1167 | 1 |
| MHCII-0000062-C | 62 | 1121 | 1 |
| MHCII-0000063-C | 63 | 1116 | 1 |
| MHCII-0000064-C | 64 | 1107 | 1 |
| MHCII-0000065-C | 65 | 1104 | 1 |
| MHCII-0000066-C | 66 | 1049 | 1 |
| MHCII-0000067-C | 67 | 1023 | 1 |
| MHCII-0000068-C | 68 | 1012 | 1 |
| MHCII-0000071-C | 69 | 941 | 1 |
| MHCII-0000073-C | 70 | 925 | 1 |
| MHCII-0000074-C | 71 | 2055 | 1 |
| MHCII-0000077-C | 72 | 859 | 1 |
| MHCII-0000078-C | 73 | 839 | 1 |
| MHCII-0000079-C | 74 | 822 | 1 |
| MHCII-0000081-C | 75 | 806 | 1 |
| MHCII-0000082-C | 76 | 793 | 1 |
| MHCII-0000084-C | 77 | 786 | 1 |
| MHCII-0000086-C | 78 | 744 | 1 |
| MHCII-0000088-C | 79 | 735 | 1 |
| MHCII-0000092-C | 80 | 696 | 1 |
| MHCII-0000096-C | 81 | 660 | 1 |
| MHCII-0000098-C | 82 | 616 | 1 |
| MHCII-0000091-C | 83 | 584 | 1 |
| MHCII-0000099-C | 84 | 584 | 1 |
| MHCII-0000101-C | 85 | 545 | 1 |
| MHCII-0000102-C | 86 | 531 | 1 |
| MHCII-0000103-C | 87 | 526 | 1 |
| MHCII-0000106-C | 88 | 493 | 1 |
| MHCII-0000109-C | 89 | 466 | 1 |
| MHCII-0000111-C | 90 | 464 | 1 |
| MHCII-0000114-C | 91 | 428 | 1 |
| MHCII-0000116-C | 93 | 398 | 1 |
| MHCII-0000118-C | 94 | 386 | 1 |
| MHCII-0000119-C | 95 | 383 | 1 |
| MHCII-0000120-C | 96 | 383 | 1 |
| MHCII-0000124-C | 97 | 341 | 1 |
| MHCII-0000125-C | 98 | 318 | 1 |
| MHCII-0000134-C | 99 | 225 | 1 |
| MHCII-0000046-C | 100 | 1741 | 1 |

**S1 Figure.** Results of randomization tests testing for MHC-based non-random mating, using average (A) and maximum (B) amino acid dissimilarity of PBS regions. Dashed vertical lines denote 95% of random distribution, whereas the solid vertical line indicate the observed mean value. An observed value located within the 95% random distribution indicates a random mating pattern.
