# Supplementary material for: Diversity of major histocompatibility complex of II B gene and mate choice in a monogamous and long-lived seabird, the Little Auk (Alle alle)
Source: PLoS One. 2024 Jun 12;19(6):e0304275. doi: 10.1371/journal.pone.0304275 (PMC11168636; doi:10.1371/journal.pone.0304275)
Supplement: S4 Table — (DOCX) [file pone.0304275.s004.docx]

**Supplementary materials**

**S4 Table.** Tajima’s D test statistics using Modified Nei-Gojorobi method with Jukes-Cantor correction. Departure from neutral selection at MHC Class IIB in Little Auks was tested within putative PBS and non-PBS regions, which were defined using Brown et al. (1993) and previous seabird genetic studies (i.e. Strandh 2011).

| **Diversity Statistic** | **PBS codons (n=32)** | **Non-PBS codons (n = 67)** |
| --- | --- | --- |
| Substitutes per synonymous site (dS) | 0.05 | 0.04 |
| Substitutions per non-synonymous site (dN) | 0.11 | 0.04 |
| $\omega$ (dN/dS) | 2.40 | 1.26 |
| z-test of selection ($\omega$ =1) | (z=2.71; p < .01) | (z=1.12; p=0.1) |

# **References**

Strandh M, Lannefors M, Bonadonna F, Westerdahl H. Characterization of MHC class I and II genes in a subantarctic seabird, the blue petrel, Halobaena caerulea (Procellariiformes). Immunogenetics. 2011;63: 653–666. doi:10.1007/s00251-011-0534-8

Brown JH, Jardetzky T, Saper MA, Samraoui B, Bjorkman PJ, Wiley DC. A hypothetical model of the foreign antigen binding site of class II histocompatibility molecules. Nature 1988; 332, 6167: 845-850. doi: [10.1038/332845a0](https://doi.org/10.1038/332845a0)
