# Supplementary material for: Diversity of major histocompatibility complex of II B gene and mate choice in a monogamous and long-lived seabird, the Little Auk (Alle alle)
Source: PLoS One. 2024 Jun 12;19(6):e0304275. doi: 10.1371/journal.pone.0304275 (PMC11168636; doi:10.1371/journal.pone.0304275)
Supplement: S1 Fig — (DOCX) [file pone.0304275.s005.docx]

**Supplementary materials**

**S1 Figure.** Results of randomization tests testing for MHC-based non-random mating, using average (A) and maximum (B) amino acid dissimilarity of PBS regions. Dashed vertical lines denote 95% of random distribution, whereas the solid vertical line indicate the observed mean value. An observed value located within the 95% random distribution indicates a random mating pattern.
