# Supplementary material for: Diversity of major histocompatibility complex of II B gene and mate choice in a monogamous and long-lived seabird, the Little Auk (Alle alle)
Source: PLoS One. 2024 Jun 12;19(6):e0304275. doi: 10.1371/journal.pone.0304275 (PMC11168636; doi:10.1371/journal.pone.0304275)
Supplement: Attachment — Submitted filename: Response to Reviewers.docx [file pone.0304275.s006.docx]

Dear Editors

Following your request and taking into account comments made by Reviewers, we revised our manuscript entitled “**Diversity of major histocompatibility complex of II B gene and mate choice in a monogamous and long-lived seabird, the Little Auk (*Alle alle*)**”. We believe that we properly addressed all the issues, including the one on possible artifacts, which was the main concern of Reviewer 1. Regarding the question of the slight overlap with two listed papers, we can ensure that no text has been copied from any external resources, and eventual overlap might be a coincidence and related to same terms and definitions used by many authors. Nevertheless, to address this specific issue, we checked the whole manuscript sentence by sentence, and modified many of them quite substantially, actually for some clarification and smooth reading. We provide our detailed responses to all of the comments, after “REPLY”. While replying and whenever relevant, we referred to clean-revised version of the manuscript, with -pasted fragments of the revised manuscript. The marked manuscript with track changes is also provided, as requested.

We are very grateful to you and the reviewers for the time and effort invested in evaluating our manuscript. We believe that our manuscript greatly improved and we are now pleased to send you the revised version and hope that you will now find it manuscript suitable for *PLOS ONE.*

Best regards,

On behalf of all the co-authors,

Katarzyna Wojczulanis-Jakubas

When submitting your revision, we need you to address these additional requirements.

1. Please ensure that your manuscript meets PLOS ONE's style requirements, including those for file naming. The PLOS ONE style templates can be found at 
https://journals.plos.org/plosone/s/file?id=wjVg/PLOSOne_formatting_sample_main_body.pdf and 
https://journals.plos.org/plosone/s/file?id=ba62/PLOSOne_formatting_sample_title_authors_affiliations.pdf

**REPLY:** We prepared the manuscript and all the associated files following the indicated templates and guidance on the PLOS ONE website.

2. We noticed you have some minor occurrence of overlapping text with the following previous publication(s) among others, which needs to be addressed:

Minias, Piotr & Pikus, Ewa & Anderwald, Dariusz. (2019). Allelic diversity and selection at the MHC class I and class II in a bottlenecked bird of prey, the White-tailed Eagle. BMC Evolutionary Biology. 19. 10.1186/s12862-018-1338-3.

Juola Frans A. and Dearborn Donald C. 2012. Sequence-based evidence for major histocompatibility complex-disassortative mating in a colonial seabird. Proc. R. Soc. B.279153–162 http://doi.org/10.1098/rspb.2011.0562

In your revision ensure you cite all your sources (including your own works), and quote or rephrase any duplicated text outside the methods section. Further consideration is dependent on these concerns being addressed.

**REPLY:** None of the part of the manuscript was copied from any other resources. Nevertheless we checked all the text and did not find any evidence of plagiarism. We believe that some text overlapping may be related to specific terms/definitions used often in a similar way by various authors. To minimize the issue, we modified the sentences throughout the whole manuscript. We also cited Juola and Donald (2012) in the revised version, to support some of our statements, as this is very relevant reference, while the manuscript was apparently missing it.

3. Please include a complete copy of PLOS’ questionnaire on inclusivity in global research in your revised manuscript. Our policy for research in this area aims to improve transparency in the reporting of research performed outside of researchers’ own country or community. The policy applies to researchers who have travelled to a different country to conduct research, research with Indigenous populations or their lands, and research on cultural artefacts. The questionnaire can also be requested at the journal’s discretion for any other submissions, even if these conditions are not met.  Please find more information on the policy and a link to download a blank copy of the questionnaire here: https://journals.plos.org/plosone/s/best-practices-in-research-reporting. Please upload a completed version of your questionnaire as Supporting Information when you resubmit your manuscript.

**REPLY:** We filled the questionnaire on inclusivity in global research and submitted it along with the revised manuscript as Supporting Information, as requested.

4. We note that the grant information you provided in the ‘Funding Information’ and ‘Financial Disclosure’ sections do not match.

When you resubmit, please ensure that you provide the correct grant numbers for the awards you received for your study in the ‘Funding Information’ section.

**REPLY:** We clarified the inconsistencies.

5. We note that you have stated that you will provide repository information for your data at acceptance. Should your manuscript be accepted for publication, we will hold it until you provide the relevant accession numbers or DOIs necessary to access your data. If you wish to make changes to your Data Availability statement, please describe these changes in your cover letter and we will update your Data Availability statement to reflect the information you provide.

**REPLY:** We have deposited all the data sequences in GenBank, accession numbers are: OR909382-OR909480. The sequences have been verified and will be published on Dec 13, 2023.

6. We note that Figure 1 in your submission contain [map/satellite] images which may be copyrighted. All PLOS content is published under the Creative Commons Attribution License (CC BY 4.0), which means that the manuscript, images, and Supporting Information files will be freely available online, and any third party is permitted to access, download, copy, distribute, and use these materials in any way, even commercially, with proper attribution. For these reasons, we cannot publish previously copyrighted maps or satellite images created using proprietary data, such as Google software (Google Maps, Street View, and Earth). For more information, see our copyright guidelines: http://journals.plos.org/plosone/s/licenses-and-copyright.
 
We require you to either (1) present written permission from the copyright holder to publish these figures specifically under the CC BY 4.0 license, or (2) remove the figures from your submission:

a. You may seek permission from the original copyright holder of Figure 1 to publish the content specifically under the CC BY 4.0 license.  
 
We recommend that you contact the original copyright holder with the Content Permission Form (http://journals.plos.org/plosone/s/file?id=7c09/content-permission-form.pdf) and the following text:
“I request permission for the open-access journal PLOS ONE to publish XXX under the Creative Commons Attribution License (CCAL) CC BY 4.0 (http://creativecommons.org/licenses/by/4.0/). Please be aware that this license allows unrestricted use and distribution, even commercially, by third parties. Please reply and provide explicit written permission to publish XXX under a CC BY license and complete the attached form.”
 
Please upload the completed Content Permission Form or other proof of granted permissions as an ""Other"" file with your submission.
 
In the figure caption of the copyrighted figure, please include the following text: “Reprinted from [ref] under a CC BY license, with permission from [name of publisher], original copyright [original copyright year].”
 
b. If you are unable to obtain permission from the original copyright holder to publish these figures under the CC BY 4.0 license or if the copyright holder’s requirements are incompatible with the CC BY 4.0 license, please either i) remove the figure or ii) supply a replacement figure that complies with the CC BY 4.0 license. Please check copyright information on all replacement figures and update the figure caption with source information. If applicable, please specify in the figure caption text when a figure is similar but not identical to the original image and is therefore for illustrative purposes only.
The following resources for replacing copyrighted map figures may be helpful:
 
USGS National Map Viewer (public domain): http://viewer.nationalmap.gov/viewer/
The Gateway to Astronaut Photography of Earth (public domain): http://eol.jsc.nasa.gov/sseop/clickmap/
Maps at the CIA (public domain): https://www.cia.gov/library/publications/the-world-factbook/index.html and https://www.cia.gov/library/publications/cia-maps-publications/index.html
NASA Earth Observatory (public domain): http://earthobservatory.nasa.gov/
Landsat: http://landsat.visibleearth.nasa.gov/
USGS EROS (Earth Resources Observatory and Science (EROS) Center) (public domain): http://eros.usgs.gov/#
Natural Earth (public domain): http://www.naturalearthdata.com/

**REPLY:** We checked type of licences for maps generated in R package *ggOceanMaps,* used by us to create the map in Fig. 1. Two parts, i.e. lands and ice polygons are from Natural Earth, which is public domain: <http://www.naturalearthdata.com>. We kept it in the maps and provided this information in the figure caption.  As bathymetry layer is not distributed under the CC BY 4.0 license (and actually not needed for the present study) we removed it from the map. If you need to confirm above the information about  copyright, please see the *ggOceanMaps* package manual, section "Citations and data sources" <https://mikkovihtakari.github.io/ggOceanMaps/>

7. Please include captions for your Supporting Information files at the end of your manuscript, and update any in-text citations to match accordingly. Please see our Supporting Information guidelines for more information: http://journals.plos.org/plosone/s/supporting-information.

**REPLY:** We modified the resubmitted files following the instructions.

**Reviewers' comments:**

**Reviewer #1:** Wojczulanis-Jakubas and colleagues present a study on MHC variation and its impact on behavior in little auks. The study is novel in its choice of system, as the species represents a rarity in avian MHC studies that are typically restricted to easier-to-study taxa and model organisms. This species is also of interest to MHC biology for being long-lived, monogamous, and, for a portion of the year, subject to the pathogen stress from extremely high population density. The authors found high diversity at the portion of the MHC they genotyped, consistent with this high pathogen-mediated selective pressure.

The portion of the paper dedicated to MHC-based mating effects is the area with the least confidence, but that is inescapable. Although the authors of the study found no evidence for disassortative mating, this may be explained (as noted by the authors) by the auks' large effective population size and extremely high MHC polymorphism, findings that will be of interest beyond the study system. The authors did, however, find evidence of significant MHC assortative mating, but concerns about distinguishing duplicated loci reduced confidence in this result. This is a weakness of the paper, but not one that diminishes all interest in the results.

**REPLY:** Thank you for this comment. We totally agree with your opinion that differentiating between loci would be of great value. However, distinguishing between duplicated loci in MHC, in general, is very challenging due to the high polymorphism in this region. One of the simplest methods that needs a lot of effort and is costly is to design PCR primers that target specific MHC alleles. This is challenging mainly due to the high sequence similarity and rarely allows assign MHC alleles to specific MHC loci (Mellinger et al. 2023 PeerJ, Vekemans et al. 2021 MolEcol, Babik 2010 MolEcolRes). Therefore, the common practice in non-model animals for which MHC genes are not well recognized is to apply a different approach. Obtained fragments of DNA/RNA may correspond to specific alleles and regions within the MHC genes and therefore they are referred to the different genetic variants within the MHC region as alleles. Such variants are used as an allele proxy (and in literature such fragments are commonly called ‘alleles’). Obviously, this is not precise but gives an opportunity for a better understanding of fragment polymorphism and provides a first step to more precise analysis. Therefore, we also decided to follow this procedure.

The introduction does a solid job of introducing a reader to the evolution of MHC, and why little auks in particular are of interest. Perhaps this addition would make the study overly topical, but I think mentioning highly pathogenic avian influenza (HPAI), which has been found in little auks and linked to migrating seabirds, would help non-specialists appreciate the importance of studying immune system evolution in this species.

**REPLY:** Thank you for this comment. We added information about the avian influenza in the lines: 76-78.

*# Relevant fragments from the manuscript:*

Lines: 76-78: “Recent outbreaks of avian influenza, particularly strongly affected seabirds [35,36], highlighting the need for deeper understanding of the functioning immune system in this group”

I have concerns about the methods used to distinguish technical artifacts from real sequence variation. If these were addressed (in many cases by just providing more detail), it would increase confidence in results.

**REPLY:** Please, find below a more detailed piece of information on artefact filtering and workflow adapted to define putative MHC II alleles.

- At line 185-186, what kind of sequencing is being performed? Sanger sequencing? More detail is needed on the method.

**REPLY:** Thank you for this comment. Indeed we overlooked to specify the description of sequencing. Below, and also in the manuscript (lines 181-185, 196-197), we improved the explanation of the method we have applied. We performed PCR with our designed set of primers for MHC II and visualized PCR products on the agarose gel. Then we selected a clear, single band (PCR product) with the targeted size, cut it off from the gel, cleaned it up, and Sanger sequenced. We compared obtained sequences to the NCBI sequences resources with nblast (Nucleotide BLAST, NCBI). Thanks to that we could check and select our fragments of interest, i.e. those that aligned with bird exon 2 MHC II fragments present in the database. Sequences we obtained had some mismatches (polymorphisms) but well corresponded to little auk closely related species.

*# Relevant fragments from the manuscript:*

Lines 181-185: “Selected fragments (ca. 300 bp) were cut off from the gel and purified with MiniElute Gel Purification Kit (Qiagen). The fragments were Sanger sequenced with specific vectorette primers. The obtained sequences were then compared to the NCBI nucleotide resources with nblast (Nucleotide BLAST, NCBI). Based on the results, we selected fragments that aligned to exon 2 MHC II and well corresponded to the Little Auk closely related species”

Lines 196-197: “Random bands were Sanger sequenced and obtained sequences were aligned in order to confirm targeted fragment (BLAST)”

- Can you provide more information on how you distinguished sequencing artifacts and real variation? Was there a similarity cutoff or did you have a cutoff based on coverage? Line 197: "We identified and removed two classes of artefacts created during PCR (point mutation and chimeras) and during next-generation sequencing (substitution and inserts)"

**REPLY:** Thank you for this very important suggestion, as we probably haven’t been enough precise about this. We clarified it now in the lines: 212-227.

## *# Relevant fragments from the manuscript:*

## Lines 212-227: “Data filtering. The processing of raw data was conducted with AmpliSAT, an online wide range of tools, for amplicon analysis, also appropriate for technology that generates single-ended reads such as Ion Torrent ([58]; http://evobiolab.biol.amu.edu.pl/amplisat/index.php). From the pipeline AmpliSAT manual, we applied: AmpliCLEAN, AmpliCHECK, AmpliSAS, and AmpliCOMPARE AmpliCOMBINE tools. Therefore, we used standardised tools for identification and filtering reads, variants and amplicons according to their similarity, coverage and frequency. We removed reads with a lower average Phred quality score (lower than 30) and reads with anomalous length (too short/too long, AmpliCLEAN). To filter out the putative alleles from artefacts we used default Ion Torrent parameters: 0.5% substitution error rate, 1% indel error rate and minimum per amplicon frequency of 1%. We also removed sequencing errors [59] and chimeras (the most abundant ones) [58,60] setting 5% frequency threshold of substitution (AmpliCHECK). To compare replicated genotyping results first, we combined multiple genotyping results (AmpliCOMBINE) and then checked for variants present in one or both compared files (AmpliCOMPARE). For retrieved alleles variant depth and other statistics were summed together from both experiments, and sequencing errors based on their low frequency and/or absence in one set of individuals were removed [53,60].”

Below we also provide a bit more elaborated response.

We used the AmpliSAT, an online wide range of tools, for amplicon analysis, also appropriate for technology that generates single-ended reads such as Ion Torrent. From the pipeline manual, we applied: AmpliCLEAN, AmpliCHECK, AmpliSAS, and AmpliCOMPARE AmpliCOMBINE tools. Detailed information on the AmpliSAT workflow is provided in the document:

<http://evobiolab.biol.amu.edu.pl/amplisat/index.php?documentation>, Sebastian et al. 2016). In general, AmpliSAT provides standardised tools for identification and filtering reads, variants and amplicons according to their similarity, coverage and frequency. Thus, to remove reads with a lower average Phred quality score (lower than 30) and reads with anomalous length (too short/too long) we used AmpliCLEAN. AmpliCHECK allowed us to fast separate reads into amplicons (i.e. each amplicon composed of all MHC sequences for the focal individuals) and to assess variants/reads frequency and depth. During this step, potential errors were also annotated. In the result file the green colour highlighted the most frequent variants. In red were highlighted variants that can be identified as sequencing errors or chimeras (the most abundant ones). We have used 5% frequency threshold to filter out the putative real alleles from artefacts. Sebastian et al. 2016 recommend to use a threshold between 3% and 25%, depending on the biological context, characteristics of the studied fragments and applied sequencing technology. Auklets MHC variability is not well-recognized therefore first we experimented with the threshold of substitution before we set it. The level of threshold was dictated by an experimentally established compromise between sensitivity and specificity in allele calling. Next, we applied AmpliSAS which is divided into three steps: 1) sequence de-multiplexing; 2) sequence clustering; and 3) artefact filtering. After de-multiplexing (updated after AmpliCHECK) each amplicon included all the MHC II sequences of a particular individual and recalculated coverage and variants frequency. Variants alignment (to each other) - during clustering - allowed the identification of more sequencing errors. Therefore, they were removed during artefact filtering. We also compared the results of AmpliCHECK and AmpliSAS.

We are aware that besides the next-generation sequencing technology allowing to retrieve and differentiate co-amplified alleles from one or more loci, it also generates reads that contain a variable number of erroneous nucleotides. We are also aware that Ion Torrent sequencing technology is susceptible to specific types of errors that can affect the accuracy of the generated sequencing data. Therefore, to improve our genotyping results for our set of samples we performed two independent PCRs (changing barcodes attached to samples in both sets of PCRs) and also we replicated sequencing. To compare replicated genotyping results we used AmpliCOMBINE and AmpliCOMPARE tools. The first tool combines multiple genotyping results. In the result file are included: 1) variants present in both compared files, 2) variants present only in the first or only in the second file (highlighted with different colours code), 3) other identified (by AmpliSAS) sequencing errors based on their low frequency. While AmpliCOMPARE annotates the differences between genotyping results from replicates, AmpliCOMBINE merges multiple genotyping results. For retrieved alleles variant depth and other statistics were summed together from both experiments.

In ion semiconductor technologies (Ion Torrent or 454 platform) one of the main sources of errors is homopolymer regions. Based on available literature it can be assumed that insertion/deletion are the most common while mismatches are between 0.18-0.34 error rate per 100 nts (Liu et al. 2012, Ross et al. 2013). Another type of false variants, such as chimeras, could be formed artificially from two or more sequences merged together. Chimeric sequences form mainly during PCR and the majority of them arise from incomplete extension during amplification. In amplicon sequencing, only a small fraction of amplicon reads is chimeric. It is estimated that usually less than 1% are typical chimeras, whereas chimeras from more than two sequences are very rare (Edgar 2016).

To summarize, to identify and exclude artifacts we took the following precautions: two independent PCRs with differently tagged set of individuals, sequencing replication, standardized pipeline for dataset analysis and manual control (along with experimental approach) of obtained results (Sommer et al. 2013, Buri et al. 2014, O’Connor et al. 2016, O’Connor et al. 2019).

We believe that we managed to eliminate the majority of possible erroneous variants. However, we cannot exclude that some putative alleles are artefacts in fact (Babik 2010). Especially since we did not follow the universally accepted rule that state that rare alleles should be present in at least two individuals (low probability that identical PCR artefacts will be generated in individuals with different genetic background, Mellinger et al. 2023). Instead, we assumed that in the auklet case (species with high Ne, Walsh et al. 2005) carefully filtered replicated variants with minimal coverage of 200 reads present in one individual could be regarded as putative alleles. Received high variance in sequences is surprising (as for non-passerine), but it also provides new insight into MHC variability in *Alcidea*. Nevertheless, we are aware that the interpretation of the results warrants caution, and further research is required. Our research provided useful tools for further research, as we generated new primers for the exon 2 MHC class II (which generate short and longer sequences of exon 2 MHC II), characterized and identified potential problems of observed variability within the studied MHC region. We think that the study of MHC with the use of other NGS platforms (especially generating long reads) will be especially valuable and may provide more precise results in the future.

- The duplication of exon 2 in some individuals (but not all) was a point of surprise for me. Two papers (refs. 67 and 68) are given as evidence of duplication of this locus in other alcids. However, are there other auk populations with polymorphism in copy number within a population? I worry it could be a technical artifact, but I would be happy to stand corrected.

**REPLY:** We addressed the question of the possible artifacts in the discussion, lines: 307-312, 348-7-354.

To our best knowledge, there are a very restricted number of papers on MHC variability/population variability in alcids. We are aware of three papers (more precisely two, as one is a master's degree thesis) published by MHC pioneers in alcids, Hollie Walsh and Vicki Friesen. And one paper published by Vasquez-Carrillo and colleagues in 2014. Therefore, we are not aware of any other population where MHC polymorphism was examined.

Within MHC, both the individual MHC genes or loci and fragments of the MHC genomic region can undergo duplications, resulting in different numbers of copies (Murphy & Weaver 2017).

Duplication of exon 2 in Major Histocompatibility Complex Class II (MHC II) genes in birds is relatively common and is one of the ways birds generate diversity in their MHC genes. Duplication leads to multiple copies of these genes or segments within MHC and enhances adaptive immune response. Such a dynamic evolution of MHC genes involves not only duplication events but also gene loss, gene conversion and different forms of recombination within and between genes. Arising or disappearing of MHC copy numbers the most probably is a stochastic process upon which selection can act (Dearborn et al. 2022, Westerdahl et al. 2022).

The macroevolutionary pattern in passerine and non-passerine 45 bird species provides new insight on the genomic structure of the MHC region (He et al. 2022). However, short-read sequencing has limited ability to reconstruct physical maps of the MHC architecture. The presence of duplicated and non-duplicated MHC genes in a single species can contribute to the overall genetic diversity of the immune system and improve the response to a wider range of pathogens (Lyons et al. 2015, Biedrzycka et al. 2017, Roved et al. 2018, Whittingham et al. 2018, Minias et al. 2019, O’Connor et al. 2019, Kaufmann 2022). For example, very interesting results were obtained for two populations of red grouse (Meyer-Lucht et al. 2016). The authors detected signatures of historical positive selection on the MHC (BLB) genes and also provided data that showed copy number variation among individuals and populations. Such diversification in MHC organization could be influenced by selection and interaction of selection and drift (Hedrick 2002, Meyer-Lucht et al. 2016). It seems that the most important factors that could shape the picture of MHC inter-individual diversity are the type of selection and its strength, the load of gene flow between populations and individuals, and effective population size. The high number of putative number alleles in auk, as we mentioned in the manuscript, could also be influenced by these factors. Our studies provide an interesting example of species (and populations) with complex and still not fully understood picture of variation/diversity in MHC organization. We hope that we have provided, in reply to the previous comment, a comprehensive explanation on filtering artefacts and putative alleles procedures. However, our results point out two main issues regarding complex MHC organization in birds: 1) not enough explored topic on intra-species diversity, and 2) the urgent need to apply long-read sequencing to obtain a bigger picture of MHC structural diversity in populations.

*# Relevant fragments from the manuscript:*

**Lines 307-312: “**We are confident the high number of detected alleles is accurate as we performed PCR in several independent sets, all samples were sequenced independently twice, obtained sequences did not resemble chimeras, and were regarded as reliable alleles if present in a high number of reads (minimum 200, in both runs). Additionally, we have applied restricted criteria to sort out sequences. We also used cDNA to confirm putative alleles and analysis of the sequence revealed no stop codons or frame shift mutations.”

**Lines 347-354: “**In some seabird populations, MHC polymorphism has also been suggested to result from ancient gene duplication events that lead to a functional genetic divergence between duplicated loci [38]. Gene duplication at MHC II B appears to be a conserved trait within many seabird clades, including Procellariformes [39,86] and alcids [71,72]. However, in most studies it remains unclear whether such duplicates are functionally identical or could be duplicated loci that have evolved and are expressed independently. While locus-specific MHC II B genotyping was outside the focus of this study, gene duplicates that have diverged and function independently can conceivably maintain high MHC polymorphism [87].**”**

- How well did the MHC alleles sequenced via RNA match those sequenced from DNA? That is, did it look like all alleles were expressed? Did you have DNA and RNA collected from the same individual to be able to do this check?

**REPLY:** We have collected RNA samples for 10 individuals, but unfortunately we hadn’t access to the DNA for these individuals. Therefore, we have no possibility to directly compare cDNA and DNA results. In the final dataset 8 individuals were included. All of them possess the most common alleles but also rare alleles. This convinced us that these rare alleles (at least part of them) are putative alleles, not artefacts. The minimal depth sequencing for the rare variant was 545 (Alle alle MHC class II antigen Alal-DRB1 gene, Alal-DRB1*085 allele, exon 2 and partial cds) and it was present in the analysed sample once (in one individual). The highest coverage for putative rare allele (Alle alle MHC class II antigen Alal-DRB1 gene, Alal-DRB1*051 allele, exon 2 and partial cds) was 2013 reads. In both cases, these alleles were obtained in two independent PCRs and sequenced twice, providing replicates for the same individual. The highest depth coverage for cDNA samples (5955 reads) was detected for Alle alle MHC class II antigen (Alal-DRB1) gene, Alal-DRB1*004 allele, exon 2 and partial cds.

We clarified the question in the lines: 112-117, 137-138, 161-163.

*# Relevant fragments from the manuscript:*

Line 112-117: “Additionally, ten birds from Greenland population were sampled *post-mortem* being a subject of another project (ADACLIM no: 388 of JF and DG). From those individuals, the spleen was collected, being removed immediately (<5 min) after birds euthanasia. The spleen was then preserved in the RNAlater (Qiagen) and stored at -20⁰C until the laboratory analysis. We used the spleen samples to amplify and sequence cDNA, to evaluate the expression status of MHC class II sequences and to identify potential pseudogenes obtained with tested primers (see the details below).”

Lines 137-138: “For further analysis based on RNA template, we performed reverse transcription in order to generate complementary DNA (cDNA).”

Lines 161-163: “In the vectorette PCR approach, we used total genomic DNA from eight individuals digested with four restriction enzymes to generate vectorette-ligated DNA templates.”

Minor suggestions:

- I would clarify at the start of the paragraph beginning on Line 108 to make it obvious that these samples are separate from the above and represent only one population. Perhaps the following would be suitable: "To evaluate the expression status of MHC class II sequences and to identify potential pseudogenes obtained with tested primers, we [additionally] amplified and sequenced cDNA [from the spleens of 10 individuals from one population.]"
**REPLY:** We clarified relevant sentences to make it clear, please see the lines: 112-117

*# Relevant fragments from the manuscript:*

Line 112-117: “Additionally, ten birds from Greenland population were sampled *post-mortem* being a subject of another project (ADACLIM no: 388 of JF and DG). From those individuals, the spleen was collected, being removed immediately (<5 min) after birds euthanasia. The spleen was then preserved in the RNAlater (Qiagen) and stored at -20⁰C until the laboratory analysis. We used the spleen samples to amplify and sequence cDNA, to evaluate the expression status of MHC class II sequences and to identify potential pseudogenes obtained with tested primers (see the details below).”

- The "Molecular sexing" paragraph occurs before any mention of DNA extraction. Please consider reordering.

**REPLY:** Thank you for this suggestion. Yes, we agree that moving the “Molecular sexing” paragraph after DNA extraction is logical.

- At line 141, I'm not sure what a "bench" of PCR primers is. Perhaps a "panel" would be better?
**REPLY:** Thank you for the comment. We have replaced “panel” with “set of” primers.

- I was surprised to see vectorette PCR used, since I thought it was not a very common technique, but I think the rationale was to design more specific primers for the species of interest? Please clarify the rationale. The paper cited as a model (ref. 50) is a Drosophila phylogeny paper from 2003. Could you explain the rationale for the selection of the method and perhaps note and cite other MHC papers that have used the same technique?

**REPLY:** We have applied vectorette PCR as it is a relatively easy and cheap technique to obtain a piece of information on not well-recognized DNA fragment of interest by extending its length from both sides. It also provides a possibility to design and test different primers for the fragment. In vectorette PCR for internal, known DNA fragment specific primer is designed, the second primer is universal (forward or reverse). That give possibility to amplify the ‘unknown’ part of sequence (contrary to standard PCR, which requires two specific primers for amplification) and extend it at one direction. In MHC research vectorette PCR was used for example by Ortman et al. 1997, Michalová et al. 2000, Babik et al. 2008, Biedrzycka & Radwan 2008, Babik et al. 2009, Zagalska-Neubauer et al. 2010, Schad et al. 2011, Kuduk et al 2012). We clarified the description of the vectorette PCR, please see the lines: 164-170.

*# Relevant fragments from the manuscript:*

Lines 164-170: “For this purpose, we adopted the modified vectorette PCR protocol from [50] as it allows to obtain information on not well-recognized DNA fragments of interest, and with success was applied in MHC research (e.g. [53–55]). In this technique, one internal primer is designed based on the available sequence, and the second primer is universal (forward or reverse). That gives a possibility to amplify the ‘unknown’ part of the sequence (contrary to standard PCR, which requires two specific primers for amplification) and extend it in one direction. Therefore, the technique provides an opportunity to design more precise primers for focal fragments.”

- You note ultimately the following: "We used the pair of primers: AukLz1 and AukRz3 for further MHC II B second exon genotyping. We also designed a separate set of primers (AukR0709, AukL0709 and AukL0809, AukR0809) to obtain longer sequences of the second and partial third exon of the MHC class II gene. However, the generated fragments were too long to use freely with Ion Torrent technology." So does this mean that you ended up only using the previously-published primers and that the specific primers you designed via vectorette PCR were not ultimately used? If so, please consider cutting the mention of vectorette PCR since it makes the methods section hard to understand. If you feel those primers are still worth publishing, perhaps the relevant methods can be put into a supplement and only briefly mentioned in the main text.

**REPLY:** Thank you for the comment, we apologize for not being clear in here. All primers we have provided in the manuscript were designed by us. We mentioned two different pairs of primers due to possible, future utilization of them. To be more precise, primers that generate longer exon 2 MHCII fragment is an option. It means that primers AukR0709, AukL0709 and AukL0809, AukR0809 were not directly used in genotyping but we provided them for researchers who are interested in generating partial exon 2 and exon 3 fragments. Instead, we have followed our ‘shorter option’, primers (AukLz1 and AukRz3) which we designed and used to amplify all auklet samples.

We have clarified this section in methods, now in a separate paragraph: “Primers development (lines: 152-197, too long to copy here). We cannot cut it off from the manuscript as it is an important methodological aspect of primer design. Please find also above (to the former comment) our reply referring directly to the vectorette PCR and its usage and utility in MHC research.

- At Line 196, Bioconductor is spelled incorrectly.

**REPLY:** Corrected as suggested.

Reviewer #2: This paper describes a study of MHC class IIb diversity in the little auk. The authors make a good case for evaluating immunogenetic diversity in typically under-studied taxa, such as seabirds. It is important to study MHC evolution across a wide range of species so that we can get a more complete picture of the way these genes evolve across vertebrates. The methods and analyses are sound. The conclusions reached were reasonable, and the authors were careful to note the limitations of the study. I think this paper is an appropriate fit for the journal. I only have minor comments, which I will list here. Provided that the authors address all reviewer feedback, I recommend this for publication.

60: Wouldn't this scenario select for assortative *or* disassortative mate choice depending on whether the female had the desirable genotype herself?

**REPLY:** We removed the possible alternative hypothesis (good gene) as we focused solely on testing the disassortative mate choice and so it could be misleading. Please see the clarified text, lines: 58-61 and 80-82.

*# Relevant fragments from the manuscript:*

Lines 58-61: “In this context, the genetic compatibility hypothesis assumes that individuals may produce MHC-heterozygous offspring if they mate disassortatively in respect to their own MHC profile (i.e. partner’s genes are supposed to be sufficiently different or complementary to own genotype) [20,25]. ”

Lines 80-82: “In this study, we focus on the Little Auk, or dovekie *Alle alle*, with the objective to characterize their population-level MHC diversity and to test whether MHC variation is maintained in this species through a disassortative mating.”

103: should "mist-nest" be "mist-net"?

**REPLY:** Corrected as suggested.

141: "was" designed

**REPLY:** Corrected.

183: delete "with"

**REPLY:** Corrected, in a way to avoid double “using” (“We performed all reactions using thermal cycler (Eppendorf) with ~~using the~~ following conditions settings…”)

188: "amplicon"

**REPLY:** Corrected as suggested.

190: "barcode"

**REPLY:** Corrected as suggested.

191: delete "of"

**REPLY:** Corrected as suggested.

241: delete the comma

**REPLY:** Corrected as suggested.

242: What was also the case? This is vague wording. Please clarify.

**REPLY:** The sentence clarified (lines 270-271): “On average two alleles per individual were recorded, both considering it in the total pool and separately for each population”

246: "remaining 26 alleles"

**REPLY:** Corrected as suggested.

273-275: I don't know if you need to summarize these numbers again, since you already did so in the results.

**REPLY:** We decided to keep this numbers as in results they are provided among other details, and here in a form of a summary, which for many readers may be the most convenient. We are happy to remove it though, if editor decides to do so.

285: "such as"

**REPLY:** Corrected as suggested.

314: delete "as a matter of fact"

**REPLY:** Corrected as suggested.

316: What about the other species you mentioned? Do they cover the breeding grounds extensively?

**REPLY:** We rewrite the sentence to convey the message we wanted to transfer, please see the lines: 338-346.

*# Relevant fragments from the manuscript:*

Lines 338-346: “MHC diversity in the Little Auk might be even greater than revealed in our study as we examined individuals from three breeding locations only. Although the sampled colonies span over a large breeding area of the species, they certainly do not cover the whole range and some colonies may have their idiosyncrasy (due to their isolation and/or habitat). High frequency of private alleles in each population and inter-population differences in frequencies of the shared alleles (Fig. 1), suggest some isolation of the breeding populations and/or adaptation to local conditions (pathogen pressure, population size, etc). Although we are not able to fully examine the inter-population differences in MHC diversity (due to limited population coverage and number of sampled individuals), the present results suggest interesting population patterns of MHC alleles, that are certainly worth exploring further.”

321: "that are certainly worth exploring further"

**REPLY:** Corrected as suggested.

Fig. 4: I'm not sure this figure needs to be in the main body of the paper because it doesn't show a significant result. Might want to add it to the supplementals.

**REPLY:** We believe the figure nicely present one of the important findings. We tested for disassortative mating and we did not find evidence for that. The figure demonstrate this message transparently and efficiently, thus we decided to keep in the main text.
