# Supplementary material for: Diversity of major histocompatibility complex of II B gene and mate choice in a monogamous and long-lived seabird, the Little Auk (Alle alle)
Source: PLoS One. 2024 Jun 12;19(6):e0304275. doi: 10.1371/journal.pone.0304275 (PMC11168636; doi:10.1371/journal.pone.0304275)
Supplement: Attachment — Submitted filename: Response to Reviewers.docx [file pone.0304275.s007.docx]

Dear Editors,

Following your request and taking into account comments made by Reviewers, we revised our manuscript entitled “**Diversity of major histocompatibility complex of II B gene and mate choice in a monogamous and long-lived seabird, the Little Auk (*Alle alle*)**”. Journal recommendation and our interpretation is that the comments were minor, basically to improve the clarity of the manuscript, which resulted in some changes in the text but not in findings and conclusions. We also noticed that comments of Reviewer2 have been made on the first version submitted to the journal (PONE-D-23-28975), while this version has been already corrected based on two other reviews (PONE-D-23-28975_R1). In result, some of the suggestions made by Reviewer 2 were no longer valid. Nevertheless, we thank the Reviewer for pointing out the places where we should pay particular attention to improve the text readability. We carefully considered all these suggestions, and if still relevant we corrected them accordingly. Following general recommendation and as a good practice we have also carefully proofread the whole manuscript, further clarifying the text. While doing so, we realized we should support some statements and we added three references (positions numbers in the reference list of the revised manuscript: 92, 93, 94, 97). We believe that all the changes we made greatly improved our manuscript.

We provide our detailed responses to all of the comments, after “REPLY”. We numbered all the replies, and sometimes used this numeration to refer to a previous reply. While replying and whenever relevant, we referred to the clean-revised version of the manuscript, with copy-pasted fragments of the revised manuscript. The marked manuscript with track changes is also provided, as requested.

We are very grateful to you and the reviewers for the time and effort invested in evaluating our manuscript. We believe that our manuscript is in a good shape and we are now pleased to send you its revised version hoping that you will now find the manuscript suitable for *PLOS ONE.*

Best regards,

On behalf of all the co-authors,

Katarzyna Wojczulanis-Jakubas

**Reviewers' comments to the Author**

**Reviewer #3:** The manuscript studies the diversity of MHC loci in little auks and hypothesize that disassortative mating may be present in the populations studied. Such observation would confirm a mechanism of genetic diversity maintenance for this loci complex as has been observed in other species. The authors establish the aims and background of the study in a complete and clear manner. They provide resources for future studies on this species and related. The previous comments by other reviewers have been addressed and the paper is very close to completion and acceptance for publication.

I only have one concern with the paper and it is the distinction between assortative mating and mate choice. Assortative (or disassortative) mating refers in the literature to the correlation in pairs concerning a particular trait, in this case the MHC genotypes. Mate choice, or preference, refers to the actual behaviour, males, females or both may exhibit when pairing. Correlation does not mean causation and this mean that the observation of assortative or disassortative mating does not necessarily mean there is mate choice. I suggest the authors to clarify this. For example, stating that whatever pattern of assortative or disassortative mating observed, may serve as proxy to identify the mate choice behaviour. The authors may find clarification and more in depth explanations in papers from the Emilio Rolán-Alvarez group (10.3389/fmars.2020.614237 and references therein for example).

**REPLY #01:** Thank you for this comment, we agree with it and believe that it occurred due to imprecise wording in our text, which we have now clarified. In the manuscript, we focused on MHC-dependent mating preferences expressed as MHC-assortative/MHC-disassortative mating which we believe might serve as a proxy explanation for mate choice. We are aware that observed MHC-dependent mating preferences do not reflect mate choice *per se* but provide potential background for mate choice patterns. Our intention was rather to identify MHC's role in mating by exploring partners similarity/dissimilarity in a fragment of MHC. Little Auks seem to be a good model for this purpose because of their high-density colonial breeding and their exposure, therefore, to potentially high pathogen pressure (as clarified this in the manuscript, lines 86-92). Our premise is that these ecological factors should clearly predict MHC-dependent mating preferences, and subsequently, indicate whether MHC variability plays a role in mate choice decisions in the Little Auk. The general assumption that MHC plays a role in mate choice in this species, however, is paradoxically made even more complicated and interesting because the Arctic environment Little Auks inhabit is believed to present a low risk of parasitemia and pathogen exposure. In summary, we incorporated changes in the manuscript to better clarify our points above, and to be more precise in our wording about MHC-dependent mating preferences in response to the reviewer comments. Lines 81-83, 87-93.

***Lines 81-83****:* “In this study, we focus on the Little Auk, or dovekie (*Alle alle*), to characterize their MHC diversity and to test whether MHC variation could be maintained in this species through MHC-dependent mating preferences.

***Lines 87-93****:* **“**Such environmental conditions could be expected to relax pathogenic selection on the MHC diversity in Little Auks. However, Little Auks also breed colonially in high densities that may facilitate pathogen transmission [27] (for example, via direct contact during sexual or aggressive interactions [47]), which in turn could increase selection pressure on MHC alleles, therefore increasing MHC diversity. These contrasting scenarios predict opposing patterns of MHC diversity in the Little Auk and complicate our understanding of the role of MHC in mating preferences and population biology of this species**”**

“

**Reviewer #4:** This manuscript presents original scientific work. It deals with a very interesting and important subject of diversity of MHC class II B gene in little auk, which is the first such investigation in the species. Although the writing is generally quite comprehensible, some parts of the manuscript are written quite unclear and sloppy, and should be rewritten. I would also suggest English editing to correct some errors and improve readability (some corrections I suggest in my comments). Additionally, there are some inconsistencies and in different sections of the manuscript (see my comments and suggestions). Here are some comments and suggestions to improve the manuscript:

**REPLY #02:** We noticed that the Reviewer’s comments have been made on the first version submitted to the journal (PONE-D-23-28975). This version has been already corrected based on two other reviews (PONE-D-23-28975_R1), so some of the suggestions are no longer valid. Nevertheless, we thank the Reviewer for pointing out the places where we should pay particular attention to improve the text readability. We considered all these suggestions, and if still relevant we corrected them accordingly. Following these and other comments, we have carefully proofread the whole manuscript and rewrote a some part of the text. We believe that the changes we incorporated according the Reviewers’ suggestions improved the manuscript.

*Introduction*

-line 53: With this context – change to In this context, but as the next sentence has the same beginning, re-write it (use synonyms)

**REPLY #03:** This part of the text has already been changed in the version of PONE-D-23-28975_R1.

-line 68: As a taxon – seabirds are a taxonomically varied group (comprising of at least 4 taxonomic orders) – so this should be changed or deleted-the authors should be consistent in writing scientific names of the species, e.g. line 77 – write Alle alle in parenthesis (and should check throughout the text)

**REPLY #04:** This part of the text has already been changed in the version of PONE-D-23-28975_R1. Please see the present version: lines: 71-81.

***Lines 71-81***: “Due to habitat preferences and some specific life-history traits, seabirds represent an interesting group to examine the functioning of the immune system, including MHC (e.g.[23,29]). Seabirds spend most of the year in the marine environment where they are exposed to an array of specific pathogens and ectoparasites [30–32], and that may differentiate them from other avian groups. In addition, many seabirds breed colonially in dense aggregations that can promote elevated transmission rates of pathogens, and the strength of selection on MHC diversity (especially in class II genes) has been shown to increase with increased coloniality and migratory behavior in birds [33]. Recent outbreaks of avian influenza have had particularly strong effects on seabirds [34,35], highlighting the need for a deeper understanding of the functioning immune system in this group. Finally, seabirds form long-term pair bonds that are critical for the successful incubation and provisioning of the brood, and thus represent a promising avenue for MHC-dependent mate-choice studies [36–39].”

-line 81 – delete „also“

**REPLY #05:** This part of the text has already been changed in the version of PONE-D-23-28975_R1.

-line 84: pathenogenic selection – should be pathogenic selection?!

**REPLY #06:** Corrected.

-Was one of the aims to compare MHC diversity of the three sampled breeding populations? If not– why are the samples divided in three groups? If yes, I would expect more results focusing on comparing them.

**REPLY #07:** We apologize for the oversight this misleading information. Our aim was to capture a MHC diversity at wider scale, so we sampled individuals from more than one breeding locations. Being aware that sampling in distant colonies potentially might show differences in MHC diversity/variation according to local habitat differences, we included information on basic differences between sampled populations (lines 109-114). However, as we primarily aimed to analyze MHC-dependent mating preferences in pairs and we have not detected significant differences in allele composition between colonies we decided not to focus on the results of the colonies’ MHC architecture. For that purpose, a greater sample size for each location would be ideal, as well as the inclusion of samples from other colonies. The present study is thus best understood as a starting point for understanding population differences in MHC diversity in the Little Auk. We corrected the paragraphs in the Introduction and Methods (lines 93-99, 121-124).

***Lines 93-99: “***In this study, we therefore genotyped MHC alleles across three main breeding locations (comprising two morphologically distingushable subspecies) to recognize MHC diversity of the species. Since selecting a mate is a critical decision with long-term fitness consequences in Little Auks (long-term pair bonds, social and genetic monogamy [48]; preference of partners with particular phenotypic traits [49]), and MHC II genotypes are known to mediate mate-choice decisions, we also tested the hypothesis that Little Auks exhibit MHC-dependent mating preferences. ***”***

***Lines 109-114: “***Spitsbergen and Greenland colonies are inhabited by the nominative subspecies *Alle a. alle*, while Franz-Josef Land is inhabited by the subspecies *Alle a. polaris.* The two subspecies are distinguished based on body size differences (the nominative subspecies is smaller) but are genetically very similar when assessed using neutral genetic markers [50]. The three colony locations sampled represent the most important breeding aggregations of the species (except for the Thule area in Northwest Greenland, [51]).***”***

***Lines 121-124***: “In summary, samples collected from all three colonies were used to characterize the general pattern of MHC diversity in the species, and the samples collected form the Spitsbergen colony were used to identify sex specific patterns of MHC diversity and potential MHC-dependent mating preferences within known breeding pairs.”

*Materials and methods*

-the authors should write more clearly what they mean by „small blood samples“ (line 96) – I suppose they collected small volumes of blood … further, the authors should add a description of the blood collecting procedure (how did they collect blood samples from auks)

**REPLY #08:** We added to the sentence information on the amount of blood we have collected (lines 104-105). Blood was collected according to a standard procedure for birds. Therefore, in our opinion, there is no need to describe the standard procedure in detail. However, we have introduced small modifications the paragraph to be more precise (lines 105-106).

lines 105-106: “…we collected small blood samples (ca. 20 µL of whole blood collected from underwing vain and preserved in 90% ethanol)”

-the authors write that they collected bird samples „across most of their breeding range“ (line 97), but in the discussion section, they write that „examined individuals … do not cover the whole breeding range of the species“ (line 316) – so I find the first sentence (line 97) a bit misleading – I suggest the authors to change the first sentence

**REPLY #09:** We deleted “most of” as we agree it was misleading and we clarified the issue (lines 93-95, 113-114)

***Lines 93-95:*** “we therefore genotyped MHC alleles across three main breeding locations (comprising two morphologically distingushable subspecies) to recognize MHC diversity of the species”

***Lines 113-114:*** “The three colony locations sampled represent the most important breeding aggregations of the species (except for the Thule area in Northwest Greenland, [51])”

-line 108 – „To evaluate the expression status of MHC class II sequences and to identify potential pseudogenes obtained with tested primers …“ – however I did not find any results regarding to that – please add those results in the manuscript (there is mention on cDNA in discussion section, but not in the results)

**REPLY #10:** The main aim of including cDNA samples was to use them as ‘a control’ for pseudogenes. cDNA results were incorporated into the general results because there were only eight cDNA samples. Our intention was not specifically to analyze little auk cDNA but to use the samples to improve allele identification. We have now clarified this in the text, please see the lines: 129-131, 160-164, 312-315. Therefore, cDNA samples were sequenced and analyzed along with DNA samples. During allele filtering, we could check for alleles identified from cDNA and confront them with other alleles obtained from DNA samples. We would then validate the repeatability and reliability of sequencing results between two sequencing sets. Both DNA and cDNA samples provide us with information on the presence of common and rare MHC alleles.

***Lines 129-131:*** “We used the spleen samples to amplify and sequence complementary DNA (e.g. cDNA), in order to control and validate the expression status of MHC class II B sequences obtained from DNA samples (see the details below).”

***Lines 160-164:*** “In the following steps of the analysis (see also below) MHC sequences obtained for cDNA samples (n = 8) were used to MHC sequences obtained from DNA samples. Based on that, we could assumed that analyzed MHC fragments represent putative, functional alleles and not non-functional pseudogenes. Therefore cDNA samples (n = 8) were used only as a control of the reliability of functionality of amplified MHC fragments.”

***Lines 312-315:*** We did not perform any formal analysis but examining alleles from the eight individuals, we could confirm the expression status of identified MHC fragments (putative alleles), generated by primers, thus MHC diversity we quantified in the Little Auk is reliable.

-the authors further describe in detail the procedure for cDNA analysis (lines 124-137) – but it ends quite abruptly (reverse transcriptase was inactivated …) – but what succeeding analysis they did with the mixture? The authors come back to cDNA at line 178 – but it is quite difficult to follow the procedures as they are discontinuously described (maybe the authors could firstly describe primer development)

**REPLY #11:** This part of the text has already been changed in the version of PONE-D-23-28975_R1. Please see the present version: lines: 151-164.

***Lines 151-164***: “For further analysis based on the RNA template, we performed reverse transcription to generate complementary DNA. In the first step (preprocessing) we added 2µl of RNA template (in total around 50ng) with 0,5 µl of 10 mM primer AukRz1 (degenerate primer, Table 1) and 10,5 µl of DEPC treated water and incubated in 65°C for 5 min, according to manufacturer’s protocol. Then we cooled the sample to room temperature. In the next step, we added 4 µl of 5x Reaction Buffer (part of RevertAid™ M-MuLV) to the preprocessed sample with 2 µl of mixed dNTP (10 mM, Thermo Scientific) and 1 µl of reverse transcriptase RevertAid™ M-MuLV, Thermo Scientific. We incubated the mixture for 1h at 42°C according to manufacturer’s protocol and the reverse transcriptase was inactivated for 10 min at 70°C.

In the following steps of the analysis (see also below) MHC sequences obtained for cDNA samples (n = 8) were used to MHC sequences obtained from DNA samples. Based on that, we could assumed that analyzed MHC fragments represent putative, functional alleles and not non-functional pseudogenes. Therefore cDNA samples (n = 8) were used only as a control of the reliability of functionality of amplified MHC fragments.”

-further, it is not clear what the authors mean by (line 178) „Based on received cDNA, we performed PCR amplification in order to receive targeted sequence of MHC.“? Please, clarify.

**REPLY #12: T**his part of the text has already been changed in the version of PONE-D-23-28975_R1. Please see present version, lines: 151-164 (see the copied fragment of manuscript above, for the reply #11)

-further, the authors mention primer aukRz1 (line 131) – here it is not clear what primer is that – at least cite Table 1 (and describe a primer – e.g. it is degenerate primer designed to anneal to ….)

**REPLY #13:** The whole paragraph on primers development clarified, see the lines: 173-183.

***Lines 173-183:*** “To characterize MHC diversity in the Little Auk, we identified and sequenced the second exon of MHC class II B genes, which is the most polymorphic segment of the peptide-biding region (PBR) [52]. First, we designed and tested a set of primers (Table 1), based on available sequences for closely related alcid species: the Razorbill (*Alca torda*) (Accession Number: EU326270.1, EU326269.1, EU326268.1), the Common Guillemot (*Uria aalge*) (Accession Number: EU326278.1, EU326276.1, EU326275.1EU326274.1), and the Atlantic Puffin (*Fratercula arctica*) (Accession Number: EU326267.1, EU326267.1, HQ822507.1, HQ822494.1). Designed degenerate or universal primers were used afterward in vectorette PCR, to obtain longer exon sequences covering the region, where we intended to locate the final primers for genotyping (Table 1 and Supplementary materials: Table S1). In the vectorette PCR, we used total genomic DNA from eight individuals digested with four restriction enzymes to generate vectorette-ligated DNA templates. “

-please correct to „according to manufacturer's protocol“ (lines 132, 136)

**REPLY #14:** Still valid for the PONE-D-23-28975_R1. Corrected as suggested.

-line 140: … we used two approaches – I think it would be better to write two phases, or two steps, to procedures … as those „approaches“ followed one after another, and they were not different approaches – it is a slight difference, but I think is important to be precise to avoid misleading the reader

**REPLY #15:** This part of the text has already been changed in the version of PONE-D-23-28975_R1. Please see the present version – all the section on the primers development has been clarified, lines: 171-220 (too much to copy paste-them here but perhaps the most important is also provided for the reply #13)

-line 147: … to design specific, degenerate primers …“ – I suppose they are those in Table 1 (although not all primers in Table 1 are degenerate), but it should be written more precisely …further, it is not clear why are all those primers in Table 1 important (maybe the authors could write in the text the 2 primers used for genotyping, and the rest of primers move to supplementary table) … further, use the term „degenerate primer“ instead of „degenerated primer“ throughout the text

**REPLY #16:** Thank you for this suggestion. We made this clearer by adding necessary information in the text and also we divided the table into two (Table 1 in the main text with the primers which generate exon 2 MHC II sequences and Supplementary material, Table S1 - with primers used during primers tests and searching procedures). Thank you for pointing out the misspelling, ‘degenerate primer’ is corrected. To see the changes please see lines: 171-220 (too much to copy paste-them here but perhaps the most important is also provided for the reply #13).

-my suggestion would be to completely move a description of vectorette PCR to supplementary materials, so that the main text is not interrupted with such detailed description, and consequently, re-writing of that part of Mat and methods section accordingly (it seems to me that this section is quite difficult to follow, so I suggest the authors re-write it in a clearer and more intelligible way)

**REPLY #17:** This part of the text has already been clarified in the previous version of the manuscript and we hope that you find the introduced improvement satisfactory. In our opinion, the vectorette PCR is an integrated part of the applied procedure of primer design. Therefore, we decided not to move its description to the supplementary materials. To see the changes on the vectorette procedure please see lines: 171-220 (too much to copy paste-them here but perhaps the most important is also provided for the reply #13).

-the authors should check citations in the text carefully, e.g. „Ko et al. 2003“ (lines 163, 169) – I suppose it should be citation 50

**REPLY #18:** This part of the text has already been clarified in the previous version of the manuscript This issue has been already addressed in PONE-D-23-28975_R1. Nevertheless, all the references have also been carefully checked now in the present version.

-lines 175-177: I am not sure what is the point of this sentence – is it important for this research? I fit is, it should be re-written to clarify it.

**REPLY #19:** The text has been already clarified in PONE-D-23-28975_R1. Please see also our reply #16. We decided to keep the information on other primers as a part of the methodology. Even if we have not used the primers after all, it is important to report we designed them and provide the reason why we did not use them. Please see the lines 207-211.

***Lines 207-211***: “The PCR product of additional pairs of primers (AukR0709, AukL0709 and AukL0809, AukR0809; Table 1) was also identified as a region of interest, i.e. the second and partial third exon of the MHC class II gene. However, the generated fragments were too long to use freely with Ion Torrent technology”

-lines 185-186: what does it mean „ … in order to confirm receiving sequence of targeted MHC“?...further, the authors should write what sequencing method was used in sequencing serviceGenomed – and describe a bit how the sequencing was performed (e.g. primers used for sequencing)

**REPLY #20:** The text has been already clarified in the PONE-D-23-28975_R1. To address what is left – primers for sequencing, see the line: 218-220.

***Lines 218-220***: “Finally, the amplified MHC fragments were Sanger sequenced using both PCR primers. For validation, the sequences containing MHC fragments were aligned and checked using the Basic Local Alignment Search Tool (BLAST).”

-in relation to that – MHC genotyping (lines 188-189) is actually sequencing again – the authors should write it

**REPLY #21:** This part of the text has already been changed in the version of PONE-D-23-28975_R1. Please see the present version: lines: 223-234.

***Lines 223-234***: “We genotyped 298-bp long amplicons of the Little Auk MHC class II B second exon with Ion Torrent Personal Genome Machine (PGM, Life Technologies, Carlsbad, CA, USA) in the Laboratory of Molecular Biology Technics at Adam Mickiewicz University in Poznań (Poland). First, we performed PCR with so-called fusion primers comprised of Ion Torrent adaptors (A adaptor and P1 adaptor), a 10-bp barcode, and our designed pair of specific primer (for PCR conditions see the section above). The unique barcodes were used to assign amplicons to individuals (Table in S1 Table). In total, a combination of 16 tagged forward primers and 16 tagged reverse primers was used to amplify individual samples (with different combinations of tags and primers). Two independent PCRs were performed on two sets of samples: 140 (all) and 126 individuals (randomly selected of the 140). Following amplification we prepared two sets of individuals pooled equimolarly and sequenced independently to estimate genotyping error. To check the quality of both sequencing data sets we used *Rqc Bioconductor* package [57].”

-line 206: Conserved MHC class II exon 2 motifs were considered as functional alleles. – the authors should explain this

**REPLY #22:** We apologize for this unfortunate statement. We clarified the sentence, please see the lines: 253-259.

***Lines 253-259***: “In total, we successfully sequenced 140 individuals (including those for which we obtained cDNA samples). Of these, 126 individuals were sequenced twice. Individuals that were sequenced only once were included in analyses if they provided at least 200 reads per allele. All sequences exhibited the correct size (298 bp) with no stop codons and no frame-shift mutations which allow exclude putative pseudogenes. They also correspond to sequences obtained for cDNA samples. Therefore, obtained MHC class II exon 2 fragments were considered putative alleles and submitted to GenBank (Accession numbers:OR909382-OR909480)”

-the authors should explain better mate-choice analysis - they should explain each „criteria“ (by the way – is that appropriate expression?

**REPLY #23:** The general statistical approach for mate choice analyses, consisting of Monte Carlo permutation tests, is available via the Manly 1997 citation and we don’t believe this method requires further clarification in the text as the randomization approach is widely used in most MHC mate-choice studies and is briefly described in Lines 292-303. However, we agree with the Reviewer’s comment (and their follow-up comment below) that the wording (i.e. “criteria” vs. “metric”) needs to be more consistent and the genetic tests better described. We are now exclusively using the word “metric” in the text to describe the four genetic variables we tested for non-random mating, and we have added sentences in the main text to better define each of these variables and why they are relevant.

Lines 292-303: “For each of the four criteria listed above, we used Monte Carlo randomization tests [69] to determine whether average observed mating patterns for each metric were significantly different from simulated expectations of random mating. To obtain random mating distributions, we resampled males and females from the study population without replacement and formed new random pairs, performing 10,000 iterations of this randomization procedure. We then compared the mean observed values for each metric (i.e. the values we calculated for naturally occurring pairs) with the distribution of randomly calculated means. We determined statistical significance by comparing the observed mean of the breeding pairs with the 2.5% and 97.5% tail of the randomized distribution of means, and Bonferroni-adjusting the alpha value by the number of randomization tests (0.05/6 = 0.008). For each metric, we assessed the likelihood of error within Monte Carlo randomization tests by iteratively decreasing the sample size and examining the effect of sample size on the consistency of obtaining a given result (for further explanation, see [70]).”

Later, the authors use the term „metric“, line 226), and/or cite the source for the method. Further, they only cite ii) criteria – MHC-band sharing coefficients – but the reference cites research using DNA fingerprinting (Wetton JH, Carter RE, Parkin DT, Walters D. Demographic study of a wild house sparrow population by DNA fingerprinting. Nature. 1987;327:147–149) – so, at least the authors should explain a bit, give more details (and is the term band sharing appropriate?).

**REPLY #24:** We now use “metric” throughout, as clarified in the reply above. The Wetton et al. 1987 citation is correct, though it is not an MHC study, but rather a foundational study on highly polymorphic genes in sparrows and how to quantify patterns in highly variable genetic markers. As such, “band-sharing” (which is also perhaps more accurately called “allele-sharing” in some studies) is just a simple calculation that quantifies the proportion of specific MHC alleles that might be shared between a breeding pair, using the equation 2F_ab_/(F_a_+F_b_). In this equation, the proportion of band sharing equals twice the number of the alleles shared between two individuals (2F_ab_), divided by the sum of uniquely different alleles each individual may have (F_a_+F_b_). We appreciate the reviewers point that this should be more clearly explained, and we have done so now in the main text, see the lines: 279-292.

***Lines 279-292***:” We used four distinct genetic metrics to test whether Little Auks select mates on the basis of MHC compatibility: i) differences in heterozygosity; ii) MHC-band sharing coefficients [68]; iii) additive amino acid differences; iv) maximum amino acid difference. Heterozygosity differences were calculated as the overall difference in MHC heterozygosity between each breeding pair, and can only be used to test whether disassortative mating occurs within a population (e.g. whether males or females are on average significantly more heterozygous at MHC II B than their partners). Band-sharing represents the proportion of unique MHC alleles shared within a breeding pair, and is calculated as twice the number of alleles shared within a pair, divided by the sum of unique alleles occurring within both individuals [(2F_ab_/(F_a_+F_b_), as per [67]]. Amino acid differences represent the number of amino acid substitutions occurring between pairs of different MHC alleles, and were quantified here as either the sum of all the differences between a pair at both loci, or the maximum difference observed between loci. We assessed these amino acid substitutions within two different contexts; i) using only the substitutions occurring within PBR regions of the MHC II B sequence, and ii)) using substitutions occurring throughout the entire fragment of the exon sequence”

Additive amino acid and maximum amino acid differences should also be explained. Further, the authors should explain how they estimated heterozygosity as they were notable to assign alleles to each of the two loci.

**REPLY #25:** Assignment of alleles to the loci in the MHC complex requires the design of locus-specific primers. We were not able to apply this approach because of the costs and time requirements of such a process. Instead, we follow the approach common for most of bird MHC publications. We clarified the text, please see the lines: 279-292 (the text fragment copy-pasted just above, as for REPLY #24).

-lines 224-225: „We assessed amino acid substitutions for both PBR regions and total exon sequences.“– It is not clearly written, should be explained better, re-written-lastly,

**REPLY #26:** We agree with the Reviewer, thank them for this comment, and have clarified this sentence with additional details. In short, we conducted randomization tests investigating the number of amino-acid substitutions that occur between the alleles of a pair using both the peptide-binding region portion of the MHC sequence (i.e. the highly variable portion most associated with gene expression resulting in olfactory cues in a variety of studies), and also the entire sequence (including the PBR region). We clarified the text, please see the lines: 279-292 (the text fragment copy-pasted just above, as for REPLY #24).

I think the authors should specify somewhere (either in Mat and methods section or in the Results) the number of individuals of each sex.

**REPLY #27:** We clarified that in the text. Please see the lines: 114-116, 118-121.

***Lines 114-116:*** “The birds sampled in Spitsbergen were breeding birds (44 pairs plus single male), captured in the nest while incubating”

***Lines 118-121:*** “The sex of the birds sampled from Greenland and Franz Josef Land was not established but given the randomized capture protocols and similar colony attendance pattern for males and females, the sex-ratio of these samples should be balanced.”

*Results*

-line 239: … 267 bp in length fragment – earlier the length of the fragment is written as 298 bp (e.g.line 205) – it is important that this discrepancy is explained in the text (further – better to write xx bp – long fragment instead of xx bp in length fragment)

**REPLY #28**: We apologize for the mistake. We have corrected the mistake and deleted “long” (lines: 223-224, 255-257, 310-311).

***Lines 223-224***: “We genotyped 298-bp long amplicons of the Little Auk MHC class II B second exon with Ion Torrent Personal Genome Machine”

***Lines 255-257***: “All sequences exhibited the correct size (298 bp) with no stop codons and no frame-shift mutations which allow exclude putative pseudogenes”

***Lines 310-311***: “We sequenced 298 bp fragments that contained 99 distinct alleles across 140 individuals”

-the authors should be consistent with the naming of populations, e.g. line 247 – omit Hornsund, write only Spitsbergen – the name written on map in Fig. 1 (as for the other two populations)

**REPLY #29:** Corrected as suggested.

-line 240: … indicating at least 2 loci (by the way – use words for numbers 1-9) and the presence of a duplicated MHC II gene fragment … - is that not the same thing? So I suggest writing „i.e.“instead of „and“

**REPLY #30:** Words for numbers 1-9 were corrected as suggested throughout the whole manuscript. We also introduced the second suggestion.

-line 241: „On average two alleles, per individual were recorded …“ (by the way, delete the comma in this part of sentence) … but in line 244 „… 56 individuals had 2 alleles (40%)…“ – so, what does it mean „on average“ in the first sentence?

**REPLY #31:** This part of the text has already been changed in the version of PONE-D-23-28975_R1. Please see the present version: lines: 316-320.

***Lines 316-320***: “A maximum of four alleles was documented within an individual, indicating at least two loci, i.e. the presence of a duplicated MHC II gene fragment, which is consistent with findings in other alcid species [71,72]. On average two alleles per individual were recorded, both within the total sample pool and separately for each population. Overall, four individuals had 4 alleles (3%), 39 individuals had 3 alleles (28%), 56 individuals had two alleles (40%), and 41 individuals had one allele (29%). “

Further, this is repetition … should be re-written.-the authors should be consistent with the names of alleles (Fig 1. and Fig. 2 – allele 1, allele 2 etc.,Table S2 – there is AS allele name and Allele name – both are different from those in Figure 1) – the authors should unify the names throughout the manuscript

**REPLY #32:** This part of the text has already been changed in the version of PONE-D-23-28975_R1. Fig1 shows alleles distribution for all the colonies, while fig 2 only for Spitsbergen (the only colony where birds were sexed) as it is with respect to sex. Then in Supplementary Table the allele names were adjusted. Please the figures and tables in the revised version.

-lines 247-251 – I would suggest that the authors write (again) the number of samples for each population, and discuss in discussion section number of alleles and number of private alleles identified per population in relation to the number of individuals analysed.

**REPLY #33:** This part of the text has already been changed in the version of PONE-D-23-28975_R1. And the sample size for each population was added, see the lines: 323-326.

***Lines 323-326:*** “The number of alleles identified per population was: in Spitsbergen - 75 alleles (64% of these were unique to the population, of n = 99), in Greenland - 20 alleles (35% unique, of n = 12) and in Franz Josef Land - 36 alleles (44% unique, of n = 29).”

-line 253: correct Table 1 to Table 2 (but if you accept my previous suggestion to move Table 1 to supplement, then this will indeed be Table 1 – of course, in that case, the authors should carefully check and correct all the tables numbers in the text).

**REPLY #34:** This has been already corrected in PONE-D-23-28975_R1. Please see also our previous responses to your comments on Table 1 (REPLY #13 and #16). We believe that both tables are meaningful and clarify the content. Therefore we decided only to modify the tables to be more precise. We also double-checked the table numbers throughout the whole text, and corrected if necessary.

-Table 2 – the authors should define „aa seqs“ -further, as the authors did not report basic polymorphism statistics separately for each population, I do not see the point in reporting them in the table as all those values could be written in a sentence.

**REPLY #35:** We thank the Reviewer for noting this unneeded confusion regarding the “aa *seqs*” in the table, and have removed it as the variable “h” is clearly defined in the table caption above and the *aa seqs* was simply our own unnecessary attempt to clarify what this meant. In regards to Table 2, we agree that the information here could be presented simply in either a small table or brief sentence format, but we prefer the table as it is more likely to be easily noticed and viewed this way.

-Fig 4 caption (line 706) - the authors should not repeat the explanation, they should just write what the figures depict (the explanation should be incorporated in the text of Results section)

**REPLY #36:** We appreciate this recommendation, but we do find this to be a stylistic matter of opinion with which we respectfully disagree. Given the complex nature of the figure, we feel it is appropriate for a detailed explanation in the caption.

-the authors should be consistent with writing the species name (in potential use of capital letters),e.g. in Table 2 they write Little auks, in line 172 they write Little Auk, but mostly it is written little auk– the authors should check and correct accordingly throughout the text, figures and tables

**REPLY #37:** The whole manuscript was checked for the issue and corrected if necessary.

-lines 259-260: … while Tajimas D analysis revealed sites with a significant excess of high-frequency segregating sites.“ – Which sites? Where those results can be found?

**REPLY #38:** Thank you, we have now added the relevant statistics as a S4 Table to provide the results of the Tajima's D test.

-lines 261-262: First sentence is redundant.

**REPLY #39:** Removed as suggested.

-lines 267: Subsequent analysis suggests … - what the authors mean by „subsequent analysis“? Did they perform some subsequent analyses? If yes, should be clearly written and explained.

**REPLY #40:** This sentence has been now clarified in the main text (lines: 343-345). A more correct was of putting it is that a subsequent realization and interpretation of the result is that the issue of having duplicated loci may (or may not be) biasing this finding of an assortative mate choice trend, and further investigation is warranted.

***Lines 343-345***: “However, this effect may be biased by the inability to distinguish between duplicated loci. Overall, insufficient evidence is available to conclude the presence of MHC-based mating effects.”

-line 703 (Fig 3 caption): „negatively selection“ should be corrected to „negative selection“

**REPLY #41:** Corrected as suggested.

*Discussion*

-lines 281-282: Was the aim of using cDNA to confirm putative alleles? Please, refer to my previous comments on cDNA – it should be clearly noted why cDNA were used, and mentioned appropriately.

**REPLY #42**: We have already corrected and developed the cDNA parts in the Methods and results (see our replies above, REPLY #10 and #11, with relevant copy-pasted fragments of the revised manuscript). In our opinion, in the discussion, we can omit this topic as cDNA samples serve as a melodic tool and we did not analyze separately cDNA samples.

-line 285: „The high similarity between alleles, as is shown in the little auk …“ – Where is that shown in the results? It should be cited again (e.g. table yy, or by repeating the values from the results). Additionally, I suppose you meant high similarity among alleles (or you had in mind two particularalleles that were very similar)? Further, are there any other explanations for the high similarity between/among alleles?

**REPLY #43:** This is invalid for the PONE-D-23-28975_R1, this part of the text has been already changed.

-lines 291-292: when making comparisons or comments, the authors should explicitly specify the values that they are commenting, like here: … can reach very high number – so please specify the numbers (like they did for non-passerine birds in the next sentence). Also, specify the MHC class that you are referring to in your discussion, at least at the beginning of the sentence.

**REPLY #44:** While editing the whole manuscript we made an effort to specifiy MHC Class II B wherever possible and taxonomy groups.

-line 295-296: the sentence is not clear, even conflicting (the little auk polymorphism is lower, but then one of the highest?) – should be re-written … further, I think the authors should put those comparisons in context of number of analysed individuals, or at least make some comment on that

**REPLY #45:** We edited the text, please see the lines: 368-370

***Lines 368-370:* “**With 2 loci per individual and a total of 99 identified MHC alleles, the Little Auk polymorphism is lower than these examples, however it remains one of the highest values reported among non-passerines and represents the highest polymorphism value detected so far among seabirds.”

-line 303: what does „then“ stand for in this sentence? What does it signify?

**REPLY #43:** This is invalid for the PONE-D-23-28975_R1, this part of the text has been already changed (roughly now lines; 371-382 but most of the text in question was simply removed)

***Lines 371-382: “***This high MHC allele diversity in the Little Auk is quite surprising given the low pathogen-abundance in the Arctic environment [42–46]. For example, no blood parasites have been detected in the Little Auk [83]. However, many MHC loci class I (but not class II) have also been detected in another polar (Antarctic) seabird, the Blue Petrel (*Halobaena caerulea*) [29]. It has been then suggested that the reason for this is that blue petrels are a colonial species that reuse underground nests, which might favor the horizontal transmission of pathogens, even if the pathogens are not that frequent in the polar environment [29]. The Little Auk is also a colonial species, yet Little Auks next above ground on cliffs or slops, and the horizontal transmission of pathogens in this Arctic environment has not been investigated. Thus, the high degree of MHC polymorphism that we identified in Little Auks may represent an ancestral polymorphism that is enhanced by a large effective population size, both in the past [51] and at present [84], and subsequently maintained by high gene flow among the breeding colonies [50,85]***”***

-line 307: „compare“ should be changed to „compared“

**REPLY #47:** This part of the text has already been changed in the version of PONE-D-23-28975_R1. (roughly now lines; 363-374 but most of the text in question was simply removed, see above the copy-pasted fragment of the manuscript, for the REPLY #43)

-line 309: „This suggests blue petrels are colonial species …“ – Does the maintenance of many MHC loci class I (but not class II) really suggest this? I think the authors should be much more attentiveon the meaning of their sentences, throughout the manuscript.

**REPLY #47: T**his part of the text has already been changed in the version of PONE-D-23-28975_R1. (roughly now lines; 363-374 but most of the text in question was simply removed, see above the copy-pasted fragment of the manuscript pasted, for the REPLY #43)

-line 317: … in frequencies of the shared alleles … should be changed to …. frequencies of the most common share alleles.

**REPLY #49:** Corrected as suggested.

-line 328: … locus -specific MHC II B genotyping was outside the focus of this study … why is it so? Could the authors comment on that in discussion?

**REPLY #50:** Locus-specific genotyping requires locus-specific primers to differentiate and amplify the respective loci. This effort was outside of the scope of the project and the genotyping approach used here, though we agree that the assignment of alleles to specific loci and an examination of expression and functionality of each locus is an ideal next step.

-lines 335-336: „While we did not investigate gene expression in this study, our evidence supports the idea that the identified alleles are functional and expressed.“ Firstly, what about cDNA analysis that you wrote you performed? Secondly, what evidence supports that identified alleles are functional? You wrote „our evidence“ so the reader would expect that it is the evidence from this investigation, but then you cite some previously published papers.

**REPLY #51:** Please see our previous reply to your previous comments regarding cDNA topic (REPLY #10 and #11).

-line 339: „… MHC genotypes, they are likely to be important components of little auk immune systems.“ – It is very well established that MHC genotypes are important to immunity. Is there something else the authors wanted to suggest?

**REPLY #52:** We are afraid that we do not exactly understand your suggestions or doubts.

-line 345: The lack of such a pattern suggests several possibilities. – This is one example of poor English writing (as far as I can tell), so the authors should consult a native speaker … maybe: There could be several reasons for the absence of such a pattern.

**REPLY #53:** Thank you for this and other suggestions. We have now carefully proofread the whole manuscript during our revision and clarified the language where it could be improved (note that one of the authors of the manuscript is a native English speaker).

-lines 351-354: The sentence starts with „… random mating….“ and ends „… little auks may select mates …“ – it is quite in conflict – should be rewritten for more clarity

**REPLY #54:** The sentence rewritten, lines: 423-426.

***Lines 423-426:*** “Finally, random mating occurring on two separate yet functionally expressed MHC II B loci may be sufficient to maintain the high degree of MHC polymorphism that we find in the population, yet Little Auks may select mates on the basis of other criteria that may only loosely be correlated with MHC, such as size or experience. “

-lines 361-365: this is quite confusing part, it is not clearly written … „effect of the same mechanism“ – which mechanism does the authors refer to?

**REPLY #54:** The text rewritten, lines: 435-440.

***Lines 435-440***: “While speculative, this perspective is supported in a broader ecological context: some studies suggest that assortative mating can mitigate sexual conflict over parental care [99]. If so, this could be particularly important dynamic in the case of the Little Auk, which require an extensive parental effort with a high degree of parental coordination during the incubation and provisioning periods, in order to successfully fledge their chicks [100–102]..”

*References*

There are some errors in references, here are some of them that I noticed:

-references 33 and 34 are very similar, but cited in a different way – the authors should check them (additionally ref 34 has repetition)

-reference 38 – repetition,

no journal name-reference 39 – please check-all references should be carefully checked once again

**REPLY #56:** Thank you for pointing this out; this issue was partially addressed in the previous revision. Nevertheless, all the references have been re-checked: the Belkin et al chapter was removed from the list, as it was found redundant (greatly overlapping with Munn et al).
