# Supplementary material for: Diversity of major histocompatibility complex of II B gene and mate choice in a monogamous and long-lived seabird, the Little Auk (Alle alle)
Source: PLoS One. 2024 Jun 12;19(6):e0304275. doi: 10.1371/journal.pone.0304275 (PMC11168636; doi:10.1371/journal.pone.0304275)
Supplement: Attachment — Submitted filename: PONE-D-23-28975_R2_Revision2_comments.docx [file pone.0304275.s008.docx]

MAJOR

First, I apologize to the authors for not being more careful in the first review, I placed my effort in what I have experience with (this is assortative mating and mate choice), and I did not pay enough attention to the materials and methods section. That is why my second review is considerably more extensive. Otherwise, I really like this study: the introduction, Results and Discussion parts are well written and are compelling. The methodology part, and specially the primer development section, albeit being confusing, show a lot of thought and careful consideration (as the authors showcase in the first paragraph of the discussion).

After reading the comments from the second reviewer, who seems to have confused the manuscript versions, I did pay more attention to the methodology section. The part that covers from the cDNA to the primer design is confusing. I hope that my comments can help the authors to simplify the text, make it easier to read, and give speed in addressing the issues. For this last part I have added text alternatives for the authors to consider.

#Introduction

No comments, the introduction is good.

#Materials and methods

The previous sections to the following look good to me, with perhaps the exception of the cDNA samples section (see below).

-Primer development and Ion-torrent sequencing

I sympathize with the authors concerning the inconsistency on the materials and methods section. In general, I consider the terms oligo, barcode, and primer to be used in very broad and confusing manners in the genetics research world. I think we would all beneficiate from being more descriptive in defining these.

I feel that the text must be more structured, and names used in a consistent manner. Preferably, following the same names as the literature that started using the Vectorette PCR approach (e.g. https://doi.org/10.1007/s00239-003-2510-x).

What I mean for more structure is to start with a paragraph which overviews all the steps required for the design of primers. Alternatively or additionally, the authors could draw a schematic figure which describes the steps. As an example of the first paragraph, you could use the following:

“The second exon of the MHC class II B genes, which is the … [52], was sequenced… . In order to do that, and since only one primer sequence is available for this genomic region (ref?), we used a vectorette PCR approach (see ref) to obtain adequate primers specific to the little auk. We use this approach given its succesful use in MHC research (refs). This procedure entails (1) to ligate a designed vectorette to the sticky ends of restriction enzyme digested DNA, (2) to perform a nested PCR where specific and vectorette primers are used and (3) to sanger-sequence resulting amplicons”

At this point I would like to ask why was it nested? Perhaps you should clarify that as well.

After that first paragraph you could first explain how you get the vectorette, the specific primer and the vectorette primer and then, and only then, describe the laboratory procedure. By the way, you do not seem to describe where are you getting the vectorette libraries from, and the ligation process of the vectorette to the digested cDNA is lacking. It could also be good to clarify how many different vectorette libraries you constructed.

At the end of the primer development section, I am afraid I do not understand the aim of the last paragraph. If you already have the primers that you need to amplify DNA and do ion-torrent sequencing, why are you making an extra amplification and using Sanger sequencing?

I am assuming that the genomic DNA used for the little auk primer discovery is the cDNA obtained for individuals described in the cDNA samples section. Is this correct? In that case, don’t say that they were only used as controls, it is a fundamental step in the primer discovery. The fact you can use them as controls as well is additionally. Be clear. You may have wanted to do so but in Lines 160 to 161 that quote does not make sense, were used to what?

I had to go through the literature to understand the process (I read the steps undertaken in https://doi.org/10.1007/s00239-003-2510-x), I would ask you, and specially for this kind of journal, not to assume that the readers are familiar with the concepts, and name and define things clearly and consistently through the text. For instance, if I am not getting it wrong, the primers you test from related species would be the specific primers (the primers that are known for the region of interest that you want to statistically analyze). You seem to be using interchangeably “specific”, “degenerate” and “internal”. If I do not get this wrong either, the universal primers would be what in the paper by Ko et al. (2003) are called vectorette primers (which is not the same as the vectorette itself from what I can understand), and in your case would be C20 and B21. Also, I am assuming that the digested genome you are using for primer discovery is the cDNA obtained from reverse transcription (see on the comments I made previously).

I believe that the authors have been very thorough to ensure good data quality and that the steps to reach it have been considered carefully and fully. Nonetheless, the text does not show that so well. If this section gets clearer, there is to my knowledge little else needed.

-Data filtering

No comments, this looks good.

-Genetic analysis

No comments, this looks good.

-Mate-choice analysis

This looks very good, well done.

-Results

No comments, looks good.

-Discussion

Line 379-382. I agree, very good point. And in general, very good discussion.

MINOR

Line 106. Vein not vain

Line 180. Afterwards not afterward

Line 184. You cannot start a new paragraph with “For this purpose”, the paragraph should be new to the previous one.

Line 184. I do not think that is the right reference, I looked it up and there was no mentioning of vectorette PCR.

I imagine that figures were sent separately and in better resolution. The ones present in the submitted revision do not look good.

Line 203-204. Again, consistency, are you sure they can be called vectorette primers? The vectorette primers you are using are C20 and B21, readers can get confused and assume that they are the same.

Line 360. Repeated “then”

Line 377. Nest instead of Next

Line 421-423. Can you clarify some more what you mean here?

Line 423-426. Good, but can you provide some reference for that minor correlation?
