# Supplementary material for: Diversity of major histocompatibility complex of II B gene and mate choice in a monogamous and long-lived seabird, the Little Auk (Alle alle)
Source: PLoS One. 2024 Jun 12;19(6):e0304275. doi: 10.1371/journal.pone.0304275 (PMC11168636; doi:10.1371/journal.pone.0304275)
Supplement: Attachment — Submitted filename: Reponse to review_rev3.docx [file pone.0304275.s009.docx]

Dear Editors,

Following your request and taking into account comments made by Reviewer #3, we revised our manuscript entitled “**Diversity of major histocompatibility complex of II B gene and mate choice in a monogamous and long-lived seabird, the Little Auk (*Alle alle*)**”. Journal recommendation and our interpretation is that the comments were minor, basically to improve the clarity of the manuscript, which resulted in some changes in the text but not in findings and conclusions. We carefully considered all these suggestions, and corrected the manuscript accordingly. Following general recommendation and as a good practice we have also carefully proofread the whole manuscript, further clarifying the text. We believe that all the changes we made greatly improved our manuscript.

We provide our detailed responses to all of the comments, after “REPLY”. While replying and whenever relevant, we referred to the clean-revised version of the manuscript, with copy-pasted fragments of the revised manuscript. The marked manuscript with track changes is also provided, as requested.

We are very grateful to you and the reviewers for the time and effort invested in evaluating our manuscript. We believe that our manuscript is in a good shape and we are now pleased to send you its revised version hoping that you will now find the manuscript suitable for *PLOS ONE.*

Best regards,

On behalf of all the co-authors,

Katarzyna Wojczulanis-Jakubas

**Reviewers' comments to the Author**

MAJOR

First, I apologize to the authors for not being more careful in the first review, I placed my effort in what I have experience with (this is assortative mating and mate choice), and I did not pay enough attention to the materials and methods section. That is why my second review is considerably more extensive. Otherwise, I really like this study: the introduction, Results and Discussion parts are well written and are compelling. The methodology part, and specially the primer development section, albeit being confusing, show a lot of thought and careful consideration (as the authors showcase in the first paragraph of the discussion).

**REPLY #1:** Thank you for finding the study well written and interesting, and thank you for you time and all the suggestion at the second round of revision. We did our best to further clarify the text, and we hope the changes are now satisfactory.

After reading the comments from the second reviewer, who seems to have confused the manuscript versions, I did pay more attention to the methodology section. The part that covers from the cDNA to the primer design is confusing. I hope that my comments can help the authors to simplify the text, make it easier to read, and give speed in addressing the issues. For this last part I have added text alternatives for the authors to consider.

**REPLY #2** As stated in the previous revision (R2; responses to Reviewers comments) the feedback of Reviewer #2, for unknown for us reasons, was based on the very first version of the manuscript. After the first round of revision (R1) and then the second one (R2) the text has greatly improved. Based on the current reviewer comments we understand that still there are some issues to clarify. We did our best to further clarify the text and we hope the changes are now satisfactory. Please see below our detailed responses (with reference to the modified manuscript).

#Introduction

No comments, the introduction is good.

**REPLY #3:** Thank you

#Materials and methods

The previous sections to the following look good to me, with perhaps the exception of the cDNA samples section (see below).

**REPLY #4:** Please find below more information and notice that we have reorganized the section on cDNA (lines 201-217).

Lines 201-217: “The complementary DNA (cDNA) samples were used to generate sequences which were regarded as a control of the reliability of functionality of MHC fragments amplified with designed specific primers. Fragments obtained from cDNA samples represent putative, functional alleles, not non-functional pseudogene. Therefore, in the following step we confronted MHC sequences for DNA and cDNA samples generated with use of final primers: AukLz1 and AukRz3 (Table 1). Finally, before performing the next-generation sequencing of all samples, to confirm target sequence (MHC II, exon 2 fragment), we also checked the PCR product (of DNA and cDNA samples) by Sanger sequencing in two directions with both final PCR primers. For validation, the sequenced fragments were aligned with use of the Basic Local Alignment Search Tool (BLAST).

To obtain cDNA we used RNA template (n = 8) and performed reverse transcription to generate cDNA. In the first step (pre-processing) we added 2µl of RNA template (in total around 50ng) with 0,5 µl of 10 mM primer AukRz1 (degenerate primer, Table 1) and 10,5 µl of nuclease-free treated water and incubated in 65°C for 5 min, according to manufacturer’s protocol. Then, we cooled the sample to room temperature. In the next step, we added 4 µl of 5x Reaction Buffer (part of RevertAid™ M-MuLV) to the preprocessed sample with 2 µl of mixed dNTP (10 mM, Thermo Scientific) and 1 µl of reverse transcriptase RevertAid™ M-MuLV, Thermo Scientific. We incubated the mixture for 1h at 42°C according to manufacturer’s protocol and the reverse transcriptase was inactivated for 10 min at 70°C.”

-Primer development and Ion-torrent sequencing

I sympathize with the authors concerning the inconsistency on the materials and methods section. In general, I consider the terms oligo, barcode, and primer to be used in very broad and confusing manners in the genetics research world. I think we would all beneficiate from being more descriptive in defining these.

**REPLY #5:** We agree that terminology in molecular papers is sometimes difficult to follow, especially in the case of terms like a primer and an oligo. Term barcodes (or tags) are restricted to the individuals’ IDs, therefore they cannot be mixed with terms primer/oligo. We understand that this is a general Reviewer impression that has not applied to our manuscript (as we do not use the term oligo and we provided a barcode definition). In most cases, it is quite easy to navigate between these terms as usually the context is very helpful (however, probably most for someone who is familiar with technical aspects of working in a molecular lab). We did our best to clarify this whole section (please see the lines: 158-199).

Lines: 158-199: “To characterize MHC diversity in the Little Auk, we identified and sequenced the second exon of MHC class II B genes, which is the most polymorphic segment of the peptide-biding region (PBR) [52]. First, we designed and tested a set of primers (Table 1 and Supplementary materials: S1 Table), based on available sequences for closely related alcid species: the Razorbill (*Alca torda*) (Accession Number: EU326270.1, EU326269.1, EU326268.1), the Common Guillemot (*Uria aalge*) (Accession Number: EU326278.1, EU326276.1, EU326275.1EU326274.1), and the Atlantic Puffin (*Fratercula arctica*) (Accession Number: EU326267.1, EU326267.1, HQ822507.1, HQ822494.1). In the next step, the designed primers along with universal ones (C20, B21, [53]) were used in vectorette PCR, to obtain longer exon sequences covering the region, where we intended to locate the final primers for genotyping (Table 1 and Supplementary materials: S1 Table). In the vectorette PCR, we used total genomic DNA from the eight individuals digested with four restriction enzymes to generate vectorette-ligated DNA templates.

For vectorette PCR, we adopted the modified vectorette PCR protocol from [53] as it allowed us to obtain information on poorly known DNA fragments of interest, and has been applied successfully in MHC research (e.g. [54–56]). In this technique, one primer (so called “internal”) is designed based on the available sequence, and the second primer is universal (C20 and B21 used as forward or reverse). This approach allows the ‘unknown’ part of the sequence to be extended in one direction and amplified (contrary to standard PCR, which requires two specific primers for amplification). Therefore, the technique provides an opportunity to design more precise, final primers for focal fragments.

First, to construct vectorette libraries, we used ca. 5 μg portion of genomic DNA which was digested with 10 U of enzymes: MunI, XapI, EcoRI, Bsu15i REs (Fermentas) at 37°C for 5 min (other steps, including ligation, were performed according [53]). Then, we have performed vectorette PCRs, where 15 μl of reaction mixture contained 7.5 μl QIAGEN Multiplex PCR Master Mix, 0.15 μM of the specific (AukLz1 or AukLz2/AukRz1, Table 1 and Supplementary materials: S1 Table) and C20 vectorette [53] primers, and 0.5 μl of vectorette-ligated digested DNA. The PCR touchdown was as follows: 95°C/15 min, 5×(94°C/30 s, 64°C/30 s, 72°C/60 s), 5×(94°C/30 s, 60°C/30 s, 72° C/60 s), 20× (94°C/30 s, 58°C/30 s, 72°C/60 s), 72°C/3min. In the second nested vectorette we used PCR 0.5 μl of product of the first PCR as template. 30 μl of PCR mixture contained 2 μL of 10× PCR buffer with (NH_4_)_2_SO_4_, 2 mM MgCl_2_, 1 μM of each primer, 0.2 mM of each dNTP and 1 U of Taq polymerase (Fermentas). In this PCR we used one specific primer (Alal1L/Alal2R/Alal3R, Supplementary materials: S1 Table) and B21 vectorette primer [53], PCR scheme was as follows: 94°C/3 min, 30× (94°C/30 s, 58°C/30 s, 72°C/60 s), 72°C/3 min. The second PCR products were run on a 1.5% agarose gel and visible fragments (ca. 300 bp) were cut off from the gel and purified with MiniElute Gel Purification Kit (Qiagen). These fragments were Sanger-sequenced with specific vectorette primers. The obtained sequences were then compared to the NCBI nucleotide resources with nblast (Nucleotide BLAST, NCBI). Based on the sequencing results, we selected fragments that aligned to MHC II B exon 2 and corresponded to sequences known from closely related auk species. Ultimately, we were able to select final primers: AukLz1 and AukRz3 for MHC II B second exon genotyping (Table 1). The PCR product of additional pairs of primers (AukR0709, AukL0709 and AukL0809, AukR0809; Supplementary materials: S1 Table) was also identified as a region of interest, i.e. the second and partial third exon of the MHC class II gene. However, generated fragments were too long to use freely with Ion Torrent technology. ”

I feel that the text must be more structured, and names used in a consistent manner. Preferably, following the same names as the literature that started using the Vectorette PCR approach (e.g. https://doi.org/10.1007/s00239-003-2510-x).

**REPLY #6:** Indeed, we agree that a part of the Methods needed to be better structured. Please find this part improved (Lines 151-224; too much to copy-paste here). What is worth to notice that we include the cDNA part in a new section (named “Primers validation”, lines: 200-224). The present structure corresponds much better to the procedures we have applied and we thank the Reviewer for this idea.

We do not understand what exactly the Reviewer had in mind writing that we should follow “the same names as the literature that started using the Vectorette PCR approach”. We used consistently throughout the whole manuscript the same terminology as mentioned in the publication (which we also have cited). However, to be more precise in the present version we added “C10, B21” in two places in the text and further clarified the whole text (lines 158-199, pasted just above, under reply #5).

What I mean for more structure is to start with a paragraph which overviews all the steps required for the design of primers. Alternatively or additionally, the authors could draw a schematic figure which describes the steps. As an example of the first paragraph, you could use the following:

“The second exon of the MHC class II B genes, which is the … [52], was sequenced… . In order to do that, and since only one primer sequence is available for this genomic region (ref?), we used a vectorette PCR approach (see ref) to obtain adequate primers specific to the little auk. We use this approach given its succesful use in MHC research (refs). This procedure entails (1) to ligate a designed vectorette to the sticky ends of restriction enzyme digested DNA, (2) to perform a nested PCR where specific and vectorette primers are used and (3) to sanger-sequence resulting amplicons”

**REPLY #7:** Thank you for the suggestion on structure with which we agree, as we mentioned just above (reply #6). We believe that the improved section helps to understand the workflow/procedures we have applied when designing and testing the primers. In our opinion present changes in the manuscript picture enough the handling of primers and their validation. Therefore, we do not agree that figures are needed as well as in our opinion there is no need to include more technical details. We would like to avoid unnecessarily prolonging the Method section and describing commonly used tools. Modifying the text we have attempted to balance the method description while parts that could cause potential misunderstanding we have expanded. For clarification and transparency of the methods, we have explained and developed the most important parts while for others we have provided additional references. As the manuscript concentrates on mate choice there is no reason to describe in detail methods that are not new and described elsewhere (and provided with an appropriate citation.

At this point I would like to ask why was it nested? Perhaps you should clarify that as well.

**REPLY #8**: We would like to note that nested PCR is a standard lab technique and we believe that there is no need to get too much into details on the techniques. The nested PCR approach eliminates spurious bands not representing the region of interest (false positive fragments), and that is obvious for researchers working with PCR techniques. Thus, if anybody would like to use our protocol, will be familiar with PCR techniques, and so there is no need to explain that in the manuscript.

After that first paragraph you could first explain how you get the vectorette, the specific primer and the vectorette primer and then, and only then, describe the laboratory procedure. By the way, you do not seem to describe where are you getting the vectorette libraries from, and the ligation process of the vectorette to the digested cDNA is lacking. It could also be good to clarify how many different vectorette libraries you constructed.

**REPLY #9**: Thank you for the suggestion, however, we cannot agree with the Reviewer that we did not provide all the above-mentioned information. Nevertheless, to better refer to methods on primer development and their validation we rearrange the subchapters and at the moment the Reviewer can find all the suggested above information included in two sections “Primer development” (subchapter slightly changed) and “Primer validation” (new subchapter).

Regarding the particular doubts (about getting the vectorette libraries, ligation and number of libraries) we have slightly modified the present version to make the information more straightforward and clear (lines 177-199). In the new subchapter (lines 201-224) we compiled information on vectorette PCR, cDNA and Sanger sequencing sections. However, all included there information was already mentioned in the previous version of the manuscript, we have mainly changed the organisation of the existing text for clarity.

Lines 177-199: „First, to construct vectorette libraries, we used ca. 5 μg portion of genomic DNA which was digested with 10 U of enzymes: MunI, XapI, EcoRI, Bsu15i REs (Fermentas) at 37°C for 5 min (other steps, including ligation, were performed according [53]). Then, we have performed vectorette PCRs, where 15 μl of reaction mixture contained 7.5 μl QIAGEN Multiplex PCR Master Mix, 0.15 μM of the specific (AukLz1 or AukLz2/AukRz1, Table 1 and Supplementary materials: S1 Table) and C20 vectorette [53] primers, and 0.5 μl of vectorette-ligated digested DNA. The PCR touchdown was as follows: 95°C/15 min, 5×(94°C/30 s, 64°C/30 s, 72°C/60 s), 5×(94°C/30 s, 60°C/30 s, 72° C/60 s), 20× (94°C/30 s, 58°C/30 s, 72°C/60 s), 72°C/3min. In the second nested vectorette we used PCR 0.5 μl of product of the first PCR as template. 30 μl of PCR mixture contained 2 μL of 10× PCR buffer with (NH_4_)_2_SO_4_, 2 mM MgCl_2_, 1 μM of each primer, 0.2 mM of each dNTP and 1 U of Taq polymerase (Fermentas). In this PCR we used one specific primer (Alal1L/Alal2R/Alal3R, Supplementary materials: S1 Table) and B21 vectorette primer [53], PCR scheme was as follows: 94°C/3 min, 30× (94°C/30 s, 58°C/30 s, 72°C/60 s), 72°C/3 min. The second PCR products were run on a 1.5% agarose gel and visible fragments (ca. 300 bp) were cut off from the gel and purified with MiniElute Gel Purification Kit (Qiagen). These fragments were Sanger-sequenced with specific vectorette primers. The obtained sequences were then compared to the NCBI nucleotide resources with nblast (Nucleotide BLAST, NCBI). Based on the sequencing results, we selected fragments that aligned to MHC II B exon 2 and corresponded to sequences known from closely related auk species. Ultimately, we were able to select final primers: AukLz1 and AukRz3 for MHC II B second exon genotyping (Table 1). The PCR product of additional pairs of primers (AukR0709, AukL0709 and AukL0809, AukR0809; Supplementary materials: S1 Table) was also identified as a region of interest, i.e. the second and partial third exon of the MHC class II gene. However, generated fragments were too long to use freely with Ion Torrent technology.“

Lines 201-224: “The complementary DNA (cDNA) samples were used to generate sequences which were regarded as a control of the reliability of functionality of MHC fragments amplified with designed specific primers. Fragments obtained from cDNA samples represent putative, functional alleles, not non-functional pseudogene. Therefore, in the following step we confronted MHC sequences for DNA and cDNA samples generated with use of final primers: AukLz1 and AukRz3 (Table 1). Finally, before performing the next-generation sequencing of all samples, to confirm target sequence (MHC II, exon 2 fragment), we also checked the PCR product (of DNA and cDNA samples) by Sanger sequencing in two directions with both final PCR primers. For validation, the sequenced fragments were aligned with use of the Basic Local Alignment Search Tool (BLAST).

To obtain cDNA we used RNA template (n = 8) and performed reverse transcription to generate cDNA. In the first step (pre-processing) we added 2µl of RNA template (in total around 50ng) with 0,5 µl of 10 mM primer AukRz1 (degenerate primer, Table 1) and 10,5 µl of nuclease-free treated water and incubated in 65°C for 5 min, according to manufacturer’s protocol. Then, we cooled the sample to room temperature. In the next step, we added 4 µl of 5x Reaction Buffer (part of RevertAid™ M-MuLV) to the preprocessed sample with 2 µl of mixed dNTP (10 mM, Thermo Scientific) and 1 µl of reverse transcriptase RevertAid™ M-MuLV, Thermo Scientific. We incubated the mixture for 1h at 42°C according to manufacturer’s protocol and the reverse transcriptase was inactivated for 10 min at 70°C.

Subset of DNA samples and all cDNA samples were used as a matrices in an amplification reaction in 21µl volume with the use of QIAGEN Multiplex PCR Kit (10 µl of 2x QIAGEN Multiplex PCR Kit, 2 µl of each 10 mM final primer and 6 µl of water). For each reaction we used a 1µl of template. We performed all reactions using a thermal cycler (Eppendorf) under the following conditions settings: 94°C for 15 min for initial denaturation followed by 34 cycles of 94°C for 30 sec, 71°C for 90 sec and 72°C for 90 sec, with additional 72°C for 10 min at the end. Obtained products were visualized by gel electrophoresis, Sanger sequenced and aligned.”

At the end of the primer development section, I am afraid I do not understand the aim of the last paragraph. If you already have the primers that you need to amplify DNA and do ion-torrent sequencing, why are you making an extra amplification and using Sanger sequencing?

**REPLY #10:** Thank you for pointing out this. We clarified this part of the Methods by reorganizing them (and better structuring). See also our comments above, at the reply #9 with the relevant manuscripts lines copy-pasted. We believe that the present structure is much clearer compared to the previous version and we appreciate your comments and suggestions.

I am assuming that the genomic DNA used for the little auk primer discovery is the cDNA obtained for individuals described in the cDNA samples section. Is this correct? In that case, don’t say that they were only used as controls, it is a fundamental step in the primer discovery. The fact you can use them as controls as well is additionally. Be clear. You may have wanted to do so but in Lines 160 to 161 that quote does not make sense, were used to what?

**REPLY #11:** We are sorry but we are not sure if we are following the Reviewer's question. It is correct that we used genomic DNA (not cDNA) for libraries construction and vectorette PCRs during the primer development. cDNA and other genomic DNA samples were used for final primers tests and final sequencing analysis (see new subchapter: “Primers validation”). cDNA was solely used for ‘final’ testing as it does not include introns and does express the products of genes (so allow exclude pseudogenes). Therefore, we have additional proof that amplified fragments are parts of active genes. Additionally, by analyzing cDNA MHC fragments we have received amplicons which we could compare to DNA amplicons and verify the subset of MHC amplicons generated for regular (DNA) samples. All above is clarified in lines 201-224 (copied for reply #9)

I had to go through the literature to understand the process (I read the steps undertaken in https://doi.org/10.1007/s00239-003-2510-x), I would ask you, and specially for this kind of journal, not to assume that the readers are familiar with the concepts, and name and define things clearly and consistently through the text. For instance, if I am not getting it wrong, the primers you test from related species would be the specific primers (the primers that are known for the region of interest that you want to statistically analyze). You seem to be using interchangeably “specific”, “degenerate” and “internal”. If I do not get this wrong either, the universal primers would be what in the paper by Ko et al. (2003) are called vectorette primers (which is not the same as the vectorette itself from what I can understand), and in your case would be C20 and B21. Also, I am assuming that the digested genome you are using for primer discovery is the cDNA obtained from reverse transcription (see on the comments I made previously).

**REPLY #12:** We appreciate that the Reviewer put a lot of effort into understanding the method. We understand that the Reviewer applies some kind of shortcut when writing “the primers you test from related species would be the specific primers”. To design and obtain specific primers we needed to perform a lot of tests on sequences of closely related species to find potential ‘specific primers’. We have noticed that the Reviewer misunderstands the idea of using the terms: “specific”, “degenerate” and “internal” primers. We do not agree that we use the terminology inconsistently. ‘Internal’ and ‘degenerate’ apply here more to technical terminology, and ‘specific’ applies to the ‘nature’ of a primer (contrary to ‘universal’). We believe that the problem with distinguishing/recognizing this specific terminology is connected with the Reviewer's area of expertise which is assortative mating and mate choice (as it was mentioned in the review). In the last review (no PONE-D-23-28975_R2) we put a lot of effort into explaining all potential misunderstandings in the method. We greatly appreciate all comments regarding the primer design and their evaluation which greatly improve the current - hopefully final - version of the manuscript. We are convenient that at the moment all the issues are explained properly, and there is no need to extend the technical part of the manuscript, especially if we cite all necessary original papers.

I believe that the authors have been very thorough to ensure good data quality and that the steps to reach it have been considered carefully and fully. Nonetheless, the text does not show that so well. If this section gets clearer, there is to my knowledge little else needed.

**REPLY #11:** Thank you. We did our best to clarify all the confusing parts you denoted.

-Data filtering

No comments, this looks good.

-Genetic analysis

No comments, this looks good.

-Mate-choice analysis

This looks very good, well done.

-Results

No comments, looks good.

-Discussion

Line 379-382. I agree, very good point. And in general, very good discussion.

**REPLY #12:** Thank you!

MINOR

Line 106. Vein not vain

**REPLY #13**: Corrected.

Line 180. Afterwards not afterward

**REPLY #14**: Corrected.

Line 184. You cannot start a new paragraph with “For this purpose”, the paragraph should be new to the previous one.

**REPLY #15**: We changed the sentence (lines 170-172) to: “For vectorette PCR, we adopted the modified vectorette PCR protocol from [53] as it allowed us to obtain information on poorly known DNA fragments of interest, and has been applied successfully in MHC research (e.g. [54–56])”

Line 184. I do not think that is the right reference, I looked it up and there was no mentioning of vectorette PCR.

**REPLY #15**: This is certainly our mistake. Thank you for spotting this! We corrected referencing to: Ko et al 2003 (now 53 in the reference list)

I imagine that figures were sent separately and in better resolution. The ones present in the submitted revision do not look good.

**REPLY #16**: We believe this is the question of the files conversion but we keep that comment in mind while examining the final proofs.

Line 203-204. Again, consistency, are you sure they can be called vectorette primers? The vectorette primers you are using are C20 and B21, readers can get confused and assume that they are the same.

**REPLY #17**: We clarified the whole section on vectorette. Please see reply 9 with relevant copy-pasted lines of the manuscript (177-199).

Line 360. Repeated “then”

**REPLY #18**: Removed

Line 377. Nest instead of Next

**REPLY #19**: Corrected

Line 421-423. Can you clarify some more what you mean here?

**REPLY #20**: Clarified: “Third, specific MHC genes may be only partially associated with a separate phenotypic trait valued by Little Auks, and thus mate choice may not operate on MHC disassortative principles at all”

Line 423-426. Good, but can you provide some reference for that minor correlation?

**REPLY #21**: Provided: Wojczulanis-Jakubas et al (2018). Assortative mating patterns of multiple phenotypic traits in a long-lived seabird. Ibis 160: 464–469. doi:10.1111/ibi.12568
